# Development and Validation of an Interpretable Machine‐Learning Model for Predicting Suboptimal Clinical Response After Sleeve Gastrectomy: A Multicenter Study

**DOI:** 10.1002/mco2.71110

**Published:** 2026-11-03

**Authors:** Yuntao Nie, An Ping, Tianshi Wang, Haibo Tang, Yishan Huang, Zhe Wang, Pengpeng Wang, Siqi Wang, Genzheng Liu, Ximing Lin, Shuman Ran, Yan Liu, Ming Zhu, Jingwei Hu, Chunhui Li, Nianrong Zhang, Baoyin Liu, Lei Zhang, Biao Zhou, Ziru Tian, Shaihong Zhu, Yinlu Ding, Hua Meng

**Affiliations:** ^1^ Department of General Surgery & Obesity and Metabolic Disease Center China‐Japan Friendship Hospital Beijing China; ^2^ School of Basic Medical Sciences Capital Medical University Beijing China; ^3^ Department of Education Beijing Luhe Hospital Capital Medical University Beijing China; ^4^ Department of General Surgery Cheeloo College of Medicine The Second Hospital of Shandong University, Shandong University Jinan Shandong China; ^5^ Department of General Surgery The Third Xiangya Hospital of Central South University Changsha Hunan China; ^6^ Department of Gastrointestinal Metabolic Surgery Zhengzhou Central Hospital, Zhengzhou University Zhengzhou Henan China; ^7^ Department of Gastrointestinal Surgery Jining No. 1 People's Hospital Jining Shandong China; ^8^ Department of Gastrointestinal Surgery The Second Affiliated Hospital of Wenzhou Medical University Wenzhou Zhejiang China; ^9^ Department of Endocrinology The Second Hospital of Hebei Medical University Shijiazhuang Hebei China; ^10^ Department of Oncology Sinopharm Tongmei General Hospital Datong Shanxi China; ^11^ National Cancer Center/National Clinical Research Center For Cancer/Cancer Hospital, Chinese Academy of Medical Science and Peking Union Medical College Beijing China

**Keywords:** machine‐learning, prediction model, sleeve gastrectomy, suboptimal clinical response, weight loss

## Abstract

Suboptimal clinical response (SCR) after sleeve gastrectomy (SG) remains a major clinical challenge, yet reliable preoperative risk stratification is lacking. Therefore, we developed and externally validated an interpretable machine‐learning model for predicting SCR after SG using multicenter data from three tertiary hospitals in China. Overall, 1312 patients were included, comprising a derivation cohort (*n* = 1094) and two external validation cohorts (*n* = 84 and *n* = 134). SCR, defined as percentage of total weight loss < 25% at 1 year, occurred in 374 (34.2%), 34 (40.5%), and 45 (33.6%) patients, respectively. Among six machine‐learning algorithms, XGB achieved the best performance. The final model incorporated eight routinely available predictors. In internal validation, the model showed excellent discrimination, with an AUC of 0.821 (95% confidence interval [CI], 0.752–0.871), sensitivity of 0.803, and specificity of 0.714. External validation confirmed satisfactory discrimination (AUC 0.761; 95% CI, 0.688–0.823), good calibration, and clinical benefit. Notably, the XGB model significantly outperformed 10 clinicians across experience levels and remained superior in clinically challenging cases. SHAP analysis enhanced interpretability, with predictor importance ranked as BMI, age, fasting glucose, type 2 diabetes, vitamin B12, calcium, total cholesterol, and gamma‐glutamyl transferase. This interpretable XGB model may enable precise preoperative risk stratification and support personalized management for patients undergoing SG.

## Graphical abstract

Suboptimal clinical response occurs in 20%–40% of patients after sleeve gastrectomy, yet reliable preoperative prediction remains challenging. We developed and externally validated an interpretable XGB model using multicenter data to identify patients at high risk of SCR. The model outperformed clinical experts and enabled personalized risk stratification, early intervention, and precision postoperative management after SG.

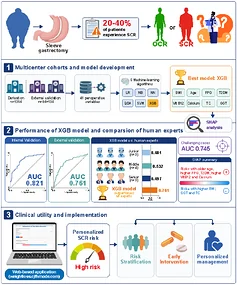

## 1 Introduction

Obesity has become a critical global health challenge, currently affecting more than 890 million adults worldwide [1]. It is a major risk factor for numerous chronic conditions, including type 2 diabetes mellitus, cardiovascular diseases, nonalcoholic fatty liver disease, and several forms of cancer [2]. In addition to its clinical implications, obesity contributes substantially to the economic burden on healthcare systems and impairs societal productivity [3]. Although lifestyle modifications and pharmacotherapy can offer some benefit, their long‐term effectiveness in achieving sustained weight loss remains limited [4]. As a result, there is an urgent need to identify effective and durable treatment strategies for obesity at the population level.

Bariatric surgery is well established as one of the most effective treatments for obesity and its associated comorbidities [5, 6]. Among these procedures, sleeve gastrectomy (SG) has gained widespread popularity due to its technical simplicity, relatively low complication rates, and favorable weight loss outcomes [7, 8]. By removing a substantial portion of the stomach, SG not only restricts caloric intake but also induces changes in gastrointestinal hormones that regulate appetite and metabolic homeostasis [9]. Despite its overall efficacy, approximately 20%–40% of patients experience a suboptimal clinical response (SCR), typically defined as inadequate weight loss [10, 11, 12]. This heterogeneity in outcomes may perpetuate obesity‐related health risks, increase the need for revisional procedures, and negatively affect psychological well‐being and quality of life [13, 14].

Identifying patients at elevated risk for SCR after SG is essential for guiding personalized treatment strategies. Although several prediction models based on clinical parameters have been proposed, many fail to account for the multifactorial and nonlinear nature of postoperative weight loss [10, 15, 16, 17, 18, 19, 20]. These models are frequently constructed using limited sets of variables and rely on conventional statistical approaches, which may be insufficient to capture the complex interactions underlying weight loss trajectories [10, 15, 16, 17, 18, 19, 20]. Moreover, their predictive accuracy is often limited, and few have undergone external validation, thus constraining their applicability in real‐world clinical settings [15, 18, 19, 20].

The rapid advancement of artificial intelligence and machine learning (ML) offers a promising avenue to address the limitations of traditional predictive models [21]. By harnessing large‐scale, multidimensional clinical datasets, ML algorithms can improve the accuracy and generalizability of outcome prediction. In this study, we developed and externally validated an ML‐based model using multicenter data to predict SCR 1 year after SG. We further compared the predictive performance of the model with that of experienced clinicians to evaluate its potential for clinical implementation. This approach aims to establish a robust framework for individualized decision‐making in bariatric surgery, promote the integration of precision medicine into routine care, and ultimately enhance long‐term outcomes for patients with obesity.

## 2 Results

### 2.1 Patient Characteristics

Overall, 1094 patients were included in the derivation cohort, with 84 patients and 134 patients included in external validation cohort 1 and external validation cohort 2, respectively, all of whom underwent SG and met the eligibility criteria (Figure 1). Among them, SCR at 1 year was observed in 374 patients (34.2%) in the derivation cohort, 34 patients (40.5%) in external validation cohort 1, and 45 patients (33.6%) in external validation cohort 2 (Table 1). The derivation cohort and the entire external validation cohort were comparable in sex, age, waist circumference, hip circumference, smoking history, alcohol consumption, hypertension, and percentage of total weight loss (%TWL) at 1 year. However, the derivation cohort had a higher body mass index (BMI) and a greater proportion of patients with type 2 diabetes mellitus (T2DM). A detailed comparison of clinical and laboratory variables between patients with SCR and those with optimal clinical response (OCR) is provided in Table S1.

**FIGURE 1:**
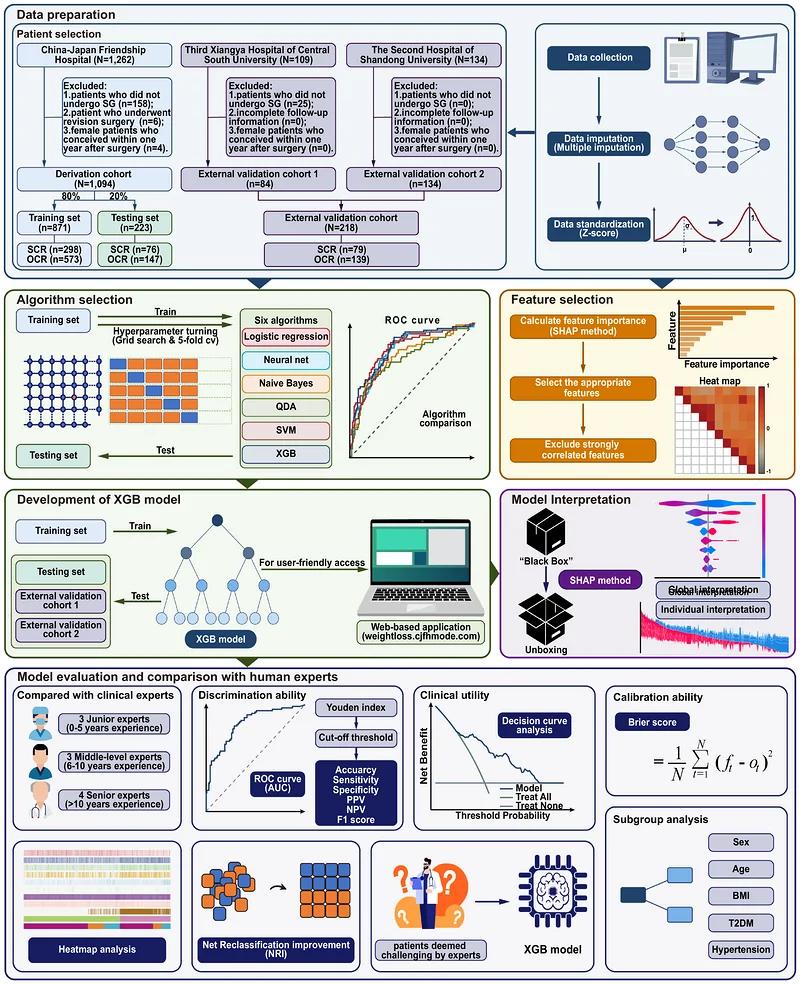
Schematic of the study workflow. AUC, area under the receiver operating characteristic curve; cv, cross validation; NPV, negative predictive value; OCR, optimal clinical response; PPV, positive predictive value; QDA, Quadratic Discriminant Analysis; ROC, receiver operating characteristic; SCR, suboptimal clinical response; SG, sleeve gastrectomy; SHAP, SHapley Additive exPlanations; SVM, Support Vector Machine; T2DM, type 2 diabetes mellitus; XGB, Extreme Gradient Boosting.

**TABLE 1:** Baseline characteristics and weight loss outcomes.

		External validation cohort			
Characteristics	Derivation cohort (*n* = 1094)	Entire external validation cohort (*n* = 218)	External validation cohort 1 (*n* = 84)	External validation cohort 2 (*n* = 134)	*p*‐value
Male, n (%)	345 (31.5)	68 (31.2)	29 (34.5)	39 (29.1)	0.984
Age, years	34.5 ± 8.8	33.3 ± 8.2	32.5 ± 8.8	33.8 ± 7.8	0.069
Height, cm	168.0 [163.0, 174.0]	167.0 [162.0, 173.0]	165.0 [160.9, 173.0]	167.50 [162.0, 172.0]	0.063
Weight, kg	108.0 [94.0, 125.0]	102.5 [90.0, 120.8]	100.8 [90.8, 118.5]	104.0 [90.0, 121.0]	0.009
BMI, kg/m^2^	38.1 [34.2, 43.0]	36.4 [32.9, 42.3]	36.3 [32.9, 40.9]	36.5 [32.9, 43.2]	0.027
Waist circumference, cm	116.0 [107.0, 128.0]	114.0 [105.0, 128.0]	112.5 [104.0, 126.0]	115.0 [106.0, 130.0]	0.148
Hip circumference, cm	121.0 [114.0, 131.0]	120.0 [110.0, 132.0]	117.0 [109.0, 128.3]	120.0 [112.0, 133.5]	0.097
Waist‐to‐hip ratio, %	96.2 ± 8.0	96.4 ± 7.4	96.9 ± 7.9	96.1 ± 7.1	0.777
Smoking history, *n* (%)	197 (18.0)	28 (12.8)	11 (13.1)	17 (12.7)	0.080
Alcohol consumption, *n* (%)	116 (10.6)	17 (7.8)	6 (7.1)	11 (8.2)	0.258
Hypertension, *n* (%)	437 (39.9)	82 (37.6)	48 (57.1)	34 (25.4)	0.571
T2DM, *n* (%)	533 (48.7)	79 (36.2)	34 (40.5)	45 (33.6)	0.001
%TWL at 1 year, %	28.7 ± 8.8	28.4 ± 1.0	27.3 ± 11.4	29.1 ± 9.2	0.649
SCR incidence, *n* (%)	374 (34.2)	79 (36.2)	34 (40.5)	45 (33.6)	0.614

*Note*: The categorical variables of the cohort data were expressed as *n* (%), and continuous variables with normal distribution were expressed as mean ± standard deviation, while medians [quartiles] were used for variables that did not satisfy normal distribution.

The *p*‐values represent the comparisons of baseline characteristics and weight loss outcomes between the derivation cohort and the entire external validation cohort.

Abbreviations: BMI, body mass index; SCR, suboptimal clinical response; T2DM, type 2 diabetes mellitus; %TWL, percentage of total weight loss.

### 2.2 Performance of the XGB Model

The predictive performance of the Extreme Gradient Boosting (XGB) model in both internal and external validation cohorts is summarized in Table 2. In the testing set, the area under the receiver operating characteristic curve (AUC) of the XGB model reached 0.821 (95% confidence interval [CI], 0.752–0.871; Figure S1A). At the optimal probability threshold determined by the Youden index (0.314), the sensitivity was 0.803 (95% CI, 0.692–0.878) and the specificity was 0.714 (95% CI, 0.629–0.784; Figure S1B). Decision curve analysis (DCA) indicated that when the predicted probability of SCR ranged from 0.15 to 0.70, the net clinical benefit of using the XGB model exceeded that of the “treat‐all” and “treat‐none” strategies (Figure S1C). In the external validation cohort, the XGB model retained satisfactory performance, with an AUC of 0.761 (95% CI, 0.688–0.823; Figure 2A), and demonstrated good calibration, as evidenced by a Brier score of 0.192 (95% CI, 0.170–0.214; Table 2). Heatmap analysis revealed that the XGB model effectively distinguished between SCR and OCR patients, exhibiting consistent performance across both the testing set and external validation cohort (Figure 3).

**TABLE 2:** Performance of the XGB model and clinical experts in the testing set and external validation cohort.

	AUC								
Model and experts	Observation (95% CI)	*p*‐value	Accuracy (95% CI)	Sensitivity (95% CI)	Specificity (95% CI)	PPV (95% CI)	NPV (95% CI)	F1 score (95% CI)	Brier score (95% CI)
**Testing set (*n* = 223)**									
XGB model	0.821 (0.752–0.871)	NA	0.744 (0.677–0.798)	0.803 (0.692–0.878)	0.714 (0.629–0.784)	0.592 (0.491–0.684)	0.875 (0.796–0.921)	0.682 (0.597–0.755)	0.171 (0.152–0.194)
**External validation cohort (*n* = 218)**									
XGB model	0.761 (0.688–0.823)	Reference	0.688 (0.624–0.743)	0.785 (0.665–0.863)	0.633 (0.552–0.707)	0.549 (0.450–0.637)	0.838 (0.758–0.897)	0.646 (0.565–0.722)	0.192 (0.170–0.214)
Average Junior experts	0.481 (0.447–0.545)	< 0.001	0.505 (0.468–0.537)	0.397 (0.348–0.451)	0.566 (0.522–0.607)	0.382 (0.279–0.490)	0.625 (0.535–0.707)	0.315 (0.260–0.381)	0.495 (0.460–0.530)
Junior expert 1	0.528 (0.493–0.571)	< 0.001	0.642 (0.564–0.697)	0.114 (0.055–0.193)	0.942 (0.893–0.973)	0.529 (0.250–0.773)	0.652 (0.583–0.712)	0.187 (0.101–0.313)	0.358 (0.281–0.417)
Junior expert 2	0.411 (0.338–0.486)	< 0.001	0.454 (0.385–0.514)	0.253 (0.163–0.359)	0.568 (0.493–0.660)	0.250 (0.159–0.355)	0.572 (0.490–0.652)	0.252 (0.156–0.348)	0.546 (0.474–0.610)
Junior expert 3	0.505 (0.445–0.557)	< 0.001	0.417 (0.349–0.482)	0.823 (0.712–0.892)	0.187 (0.124–0.254)	0.365 (0.299–0.439)	0.650 (0.479–0.781)	0.506 (0.422–0.576)	0.583 (0.513–0.647)
Average middle‐level experts	0.532 (0.493–0.576)	< 0.001	0.607 (0.556–0.656)	0.257 (0.192–0.334)	0.806 (0.758–0.845)	0.452 (0.316–0.591)	0.657 (0.589–0.725)	0.303 (0.232–0.387)	0.393 (0.343–0.442)
Middle‐level expert 1	0.490 (0.429–0.558)	< 0.001	0.541 (0.472–0.601)	0.304 (0.203–0.405)	0.676 (0.595–0.742)	0.348 (0.237–0.472)	0.631 (0.551–0.703)	0.324 (0.235–0.423)	0.459 (0.388–0.522)
Middle‐level expert 2	0.586 (0.526–0.653)	< 0.001	0.642 (0.573–0.702)	0.380 (0.277–0.494)	0.791 (0.720–0.849)	0.508 (0.367–0.638)	0.692 (0.614–0.758)	0.435 (0.324–0.541)	0.358 (0.289–0.417)
Middle‐level expert 3	0.519 (0.487–0.557)	< 0.001	0.638 (0.569–0.697)	0.089 (0.038–0.171)	0.950 (0.901–0.976)	0.500 (0.179–0.727)	0.647 (0.583–0.720)	0.151 (0.069–0.273)	0.362 (0.302–0.422)
Average senior experts	0.497 (0.470–0.530)	< 0.001	0.580 (0.529–0.624)	0.196 (0.151–0.247)	0.799 (0.764–0.834)	0.379 (0.277–0.484)	0.636 (0.566–0.704)	0.244 (0.190–0.307)	0.420 (0.373–0.470)
Senior expert 1	0.554 (0.517–0.608)	< 0.001	0.661 (0.583–0.720)	0.165 (0.096–0.264)	0.942 (0.892–0.972)	0.619 (0.381–0.800)	0.665 (0.596–0.731)	0.260 (0.148–0.370)	0.339 (0.275–0.404)
Senior expert 2	0.439 (0.390–0.485)	< 0.001	0.532 (0.463–0.596)	0.101 (0.045–0.181)	0.777 (0.696–0.841)	0.205 (0.091–0.344)	0.603 (0.528–0.675)	0.136 (0.059–0.244)	0.468 (0.399–0.532)
Senior expert 3	0.522 (0.461–0.588)	< 0.001	0.564 (0.486–0.619)	0.367 (0.257–0.479)	0.676 (0.593–0.750)	0.392 (0.281–0.500)	0.653 (0.573–0.730)	0.379 (0.286–0.485)	0.436 (0.362–0.500)
Senior expert 4	0.475 (0.426–0.528)	< 0.001	0.564 (0.495–0.624)	0.152 (0.083–0.241)	0.799 (0.721–0.859)	0.300 (0.174–0.447)	0.624 (0.551–0.694)	0.202 (0.118–0.302)	0.436 (0.368–0.500)

*Note*: The *p*‐values refer to the comparison of the AUC between XGB model and experts through bootstrapping with 1000 resamples.

Abbreviations: AUC, area under the receiver operating characteristic curve; NA, not applicable; NPV, negative predictive value; PPV, positive predictive value.

**FIGURE 2:**
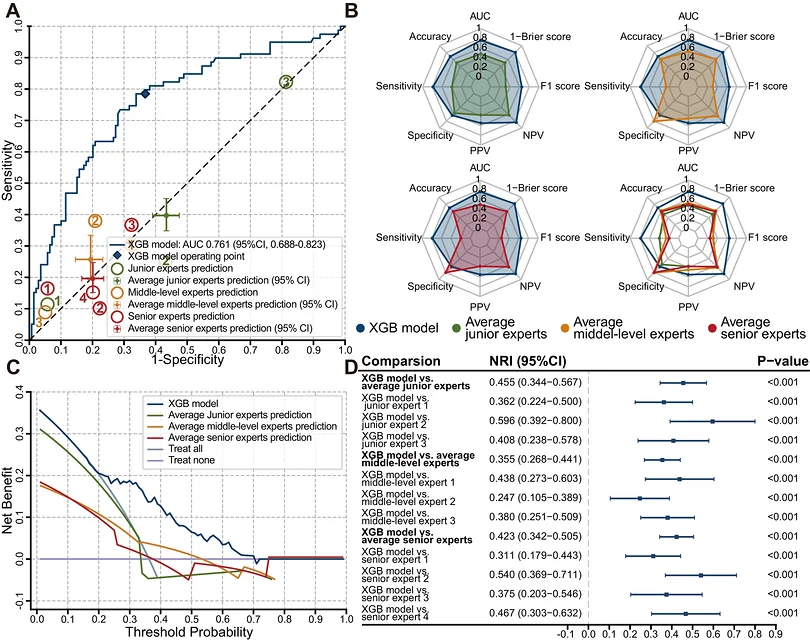
Performance of the XGB model in the external validation cohort and comparison with human experts. (A) Discrimination of the XGB model by ROC curve analysis. The operating point was determined by the Youden index. Each small colored circle represents the sensitivity and specificity of an individual expert, with error bars illustrating the 95% CI for the average sensitivity and specificity of three groups of experts. (B) The performance of the XGB model was compared with that of three groups of experts across multiple metrics. (C) Decision curve analysis comparing the net benefit of the XGB model with that of three groups of experts across a range of threshold probabilities. (D) The categorical NRI was calculated to assess the improvement in predictive accuracy of the model compared with expert judgment. AUC, area under the receiver operating characteristic curve; CI, confidence interval; NPV, negative predictive value; NRI, net reclassification improvement; PPV, positive predictive value.

**FIGURE 3:**
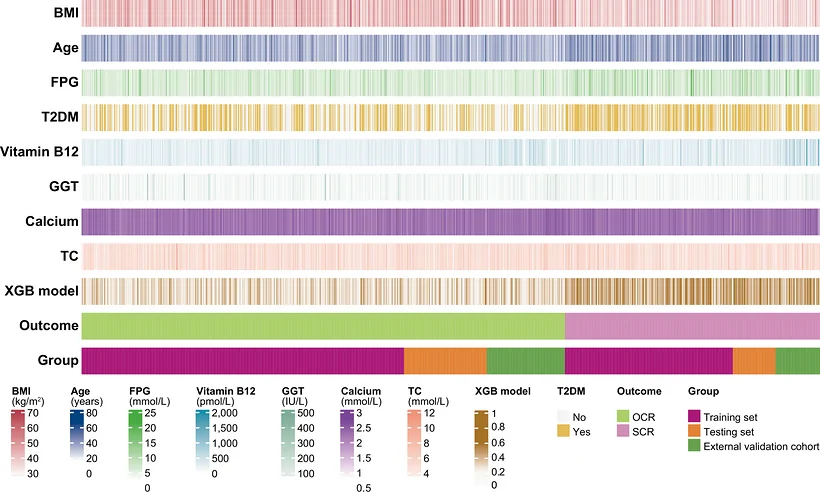
Heatmap of patient characteristics and XGB model predictions. The rows represent a range of clinical features, including BMI, age, FPG, type 2 diabetes mellitus status, vitamin B12 levels, GGT, calcium, and TC, along with XGBoost model predictions, outcomes (OCR or SCR), and group assignments (training set, testing set, and external validation cohort). The columns correspond to individual samples. Color intensity indicates the values of each feature, with distinct color scales used for each feature. BMI, body mass index; FPG, fasting plasma glucose; GGT, gamma glutamyl transferase; OCR, optimal clinical response; SCR, suboptimal clinical response; SHAP, SHapley Additive exPlanations; TC, total cholesterol; T2DM, type 2 diabetes mellitus.

In both the testing set and external validation cohorts, the performance of the XGB model remained robust across different subgroups (Table S2).

### 2.3 Comparison With Human Experts

The prediction results from 10 human experts are shown in Figure S2. The intraclass correlation coefficients (ICCs) for judgments by doctors with varying levels of experience were all below 0.3, indicating poor inter‐rater reliability among the experts. Table 2 summarizes the diagnostic metrics of the XGB model and all human experts in the external validation cohort. The AUC of the XGB model was significantly higher than the average AUC of junior experts (0.481 [95% CI, 0.447–0.545], *p* < 0.001), middle‐level experts (0.532 [95% CI, 0.493–0.576], *p* < 0.001), and senior experts (0.497 [95% CI, 0.470–0.530], *p* < 0.001), and also exceeded the performance of each individual expert (Figure 2A). In addition, the diagnostic metrics and clinical utility (Figure 2B,C) of the XGB model were all superior to those of the human experts. All experts demonstrated a statistically significant and negative net reclassification improvement (NRI) relative to the XGB model, indicating inferior performance in distinguishing SCR from OCR (Figure 2D).

The diagnostic performance of the XGB model also surpassed that of human experts in both external validation cohort 1 (Table S3 and Figure S3) and external validation cohort 2 (Table S4 and Figure S4). In the group of patients defined as “challenging cases,” the XGB model achieved a stable AUC of 0.745 (95% CI, 0.598–0.852), consistently outperforming all experts with statistical significance (*p* < 0.05 for all) (Table S5 and Figure S5).

### 2.4 Model Interpretation

As shown in Figure 4A, the contribution of each feature in the XGB model was evaluated using average SHapley Additive exPlanations (SHAP) values and ranked in descending order. BMI, age, fasting plasma glucose (FPG), and T2DM were identified as the four most influential predictors. In Figure 4B, older age, higher FPG, presence of T2DM, elevated vitamin B12 levels, and increased calcium levels were associated with a higher likelihood of SCR, whereas higher BMI, elevated gamma‐glutamyl transferase (GGT), and increased total cholesterol (TC) were associated with OCR. The actual values versus SHAP values of the eight selected features are shown in Figure S6, where SHAP values greater than zero indicate a positive contribution toward the prediction of SCR. For instance, patients with BMI ≤ 39.29 kg/m^2^, age ≥ 36 years, FPG ≥ 6.37 mmol/L, T2DM, VitB12 ≥ 321.78 pmol/L, GGT ≤ 35.72 IU/L, Ca ≥ 2.40 mmol/L, and TC ≤ 4.90 mmol/L had SHAP values above zero, thus shifting the model prediction toward the SCR category.

**FIGURE 4:**
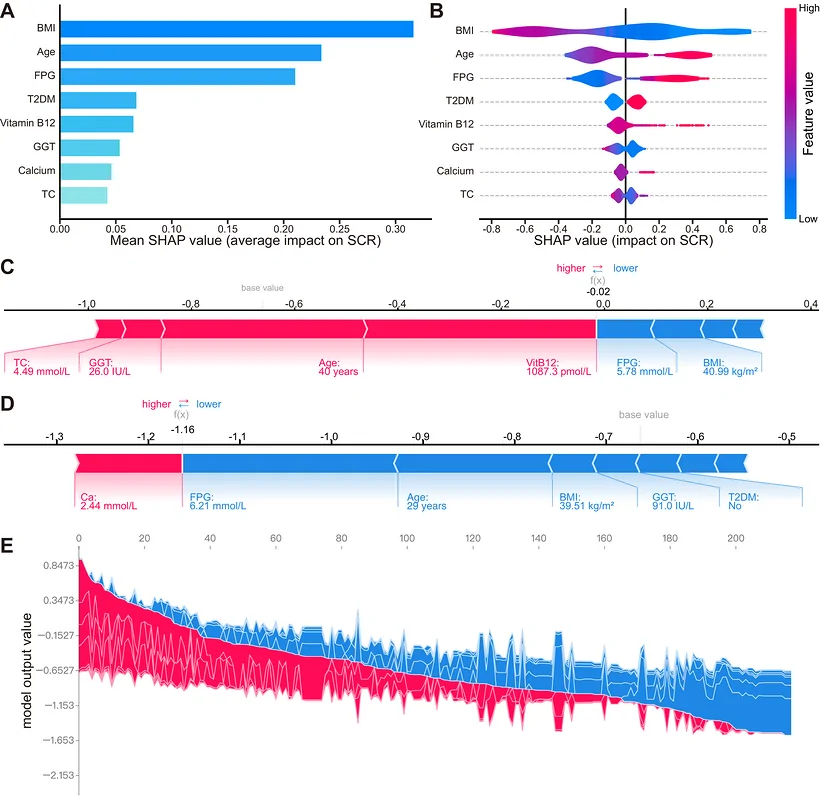
SHAP algorithm for XGB model interpretation. (A) Feature importance plot of the XGB model, ranking the eight features by their impact on the model's predicted probability, from highest to lowest. (B) SHAP summary dot plot, showing the contribution of each feature. The blue and red points in each row represent patients having low to high values of the specific feature, while the x‐axis shows the SHAP value, indicating the impact on the model. (C, D) Force plots for individual patients illustrating the contribution of each feature to the output of XGB model. (C) and (D) represent two patients with high and low risk of SCR, respectively. The red sections denote risk factors, while the blue sections indicate protective factors. (E) Force plot for the external validation cohort. Each patient is shown along the x‐axis, while the features’ contributions were represented by the y‐axis: an increased red section for each individual patient represented a greater probability toward the decision of SCR. BMI, body mass index; FPG, fasting plasma glucose; GGT, gamma glutamyl transferase; SCR, suboptimal clinical response; SHAP, SHapley Additive exPlanations; TC, total cholesterol; T2DM, type 2 diabetes mellitus.

To further enhance interpretability, local explanations were generated using SHAP force plots, demonstrating how predictions were derived for individual patients. Figure 4C displays the feature contributions for a patient who developed SCR after SG, where the value of age, vitamin B12, GGT, and TC contributed to the prediction of SCR. Figure 4D illustrates the feature contributions for a patient with actual OCR. In addition, Figure 4E presents SHAP explanations for patients in the external validation cohort.

### 2.5 User‐Friendly Application for Clinical Utility

For improved accessibility, the XGB model was deployed as a web‐based application (Figure 5). Upon entering the relevant input values, the application automatically generates the predicted risk of SCR for an individual patient. In addition, a personalized force plot is displayed to illustrate the contribution of each feature to the prediction. The application is publicly available at: weightloss.cjfhmodel.com.

**FIGURE 5:**
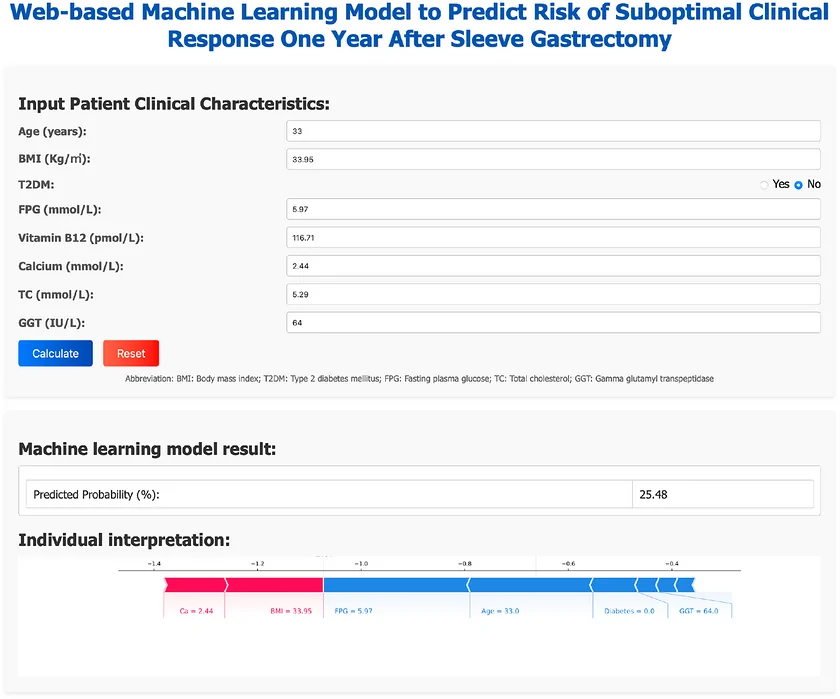
Screenshot of the web application. Screenshot of the web application, which is available at weightloss.cjfhmodel.com. The interface accepts inputs of patient clinical data, including age, BMI, type 2 diabetes mellitus, FPG, vitamin B12, calcium, TC, GGT. Upon submission, the model provides a probability of SCR. Additionally, a SHAP force plot shows the impact of each feature on the prediction, helping doctors assess the influence of various patient characteristics. In the SHAP force plot, “Diabetes = 0.0” represents non‐T2DM. BMI, body mass index; FPG, fasting plasma glucose; GGT, gamma glutamyl transferase; TC, total cholesterol; T2DM, type 2 diabetes mellitus.

## 3 Discussion

In this study, we developed an ML‐based prediction model using multicenter cohort data to estimate the risk of SCR one year after SG. The model demonstrated strong discrimination and calibration in both internal and external validation cohorts. Notably, it significantly outperformed human experts with varying levels of clinical experience, underscoring its clinical utility and potential to enhance individualized decision‐making.

SCR occurs in approximately 20%–40% of patients following SG [10, 11, 12], posing a persistent challenge in clinical practice. Despite its clinical importance, a standardized definition of SCR remains lacking [22]. Recent evidence suggests that %TWL < 25% provides superior sensitivity and specificity over alternative thresholds, improving the identification of patients with truly inadequate weight loss [22]. This cut‐off is also less influenced by baseline BMI [23, 24] and remains strongly associated with the presence and resolution of metabolic syndrome [19, 25]. Based on this evidence, we adopted %TWL < 25% as the definition of SCR in this study. Using this criterion, the incidence of SCR was 34.2% in the derivation cohort and 36.2% in the external validation cohort, consistent with prior reports.

Although several SCR prediction models have been proposed, most have substantial limitations. Many were developed using small sample sizes and conventional statistical methods (e.g., logistic regression) [10, 15, 16, 17, 18, 19, 20], which are often unable to capture nonlinear relationships [26], resulting in limited predictive performance. Moreover, external validation is frequently lacking [15, 18, 19, 20], restricting generalizability. Some models rely on postoperative variables, limiting their use for preoperative risk stratification [15, 17]. In contrast, our model was built on a large derivation cohort using six commonly applied ML algorithms, with XGB selected based on superior performance. XGB has been shown to effectively capture complex interactions and has demonstrated robust predictive ability across clinical domains [27]. We conducted rigorous external validation in two independent cohorts, where the XGB model achieved an AUC of 0.761 (95% CI, 0.688–0.823), closely aligned with its internal performance (AUC, 0.821 [95% CI, 0.752–0.871]), indicating strong generalizability. Importantly, only routinely collected clinical and laboratory variables were used [28], ensuring the applicability of the model in preoperative settings. The final model included eight readily available predictors, supporting its feasibility for integration into routine workflows.

To our knowledge, this is the first study to compare SCR prediction model performance directly against expert clinical judgment. Remarkably, we observed poor interrater reliability among experts (ICC < 0.3), and their predictive accuracy was suboptimal (AUCs ∼ 0.5), regardless of experience level. These findings highlight the difficulty clinicians face in accurately identifying high‐risk patients based solely on clinical intuition and general evidence. Although clinicians may consider factors such as dietary habits, psychological status, and adherence, these are often highly variable and difficult to quantify. In this study, expert predictions were based on the same 41 objective variables available to the model. Nonetheless, the XGB model consistently outperformed all experts, including those with extensive experience, and maintained superior performance even in cases deemed difficult by clinicians. The significantly positive NRI further supports the enhanced predictive accuracy of the model over expert judgment, enabling more precise risk stratification.

Our findings identified several clinical predictors associated with SCR. Older age, elevated FPG, T2DM, higher vitamin B12, and higher calcium levels were positively associated with SCR, whereas higher BMI, GGT, and TC were associated with OCR. Prior studies suggest that individuals with higher BMI may experience more extensive gastric resection and greater suppression of ghrelin [29, 30], resulting in enhanced appetite reduction [31]. Older age may contribute to SCR due to lower physical activity [32], longer disease duration [33], and entrenched lifestyle behaviors [34]. Elevated FPG and T2DM may impair glucagon‐like peptide‐1 (GLP‐1) receptor signaling, leading to reduced appetite control and metabolic dysregulation [35, 36, 37]. Higher GGT and TC likely reflect greater visceral adiposity [38, 39], which is more responsive to surgical fat loss [40], contributing to better weight outcomes. The associations of vitamin B12 and calcium with SCR may reflect underlying pathophysiologic mechanisms not yet fully understood and warrant further investigation.

This study presents several important clinical implications. The XGB model demonstrated significantly superior discrimination and sensitivity compared with human experts, enabling accurate preoperative identification of patients at high risk for SCR following SG. By providing individualized risk estimates, the model may complement clinical judgment and facilitate more informed patient counseling and shared decision‐making. Specifically, clinicians could use the predicted risk to communicate the likelihood of achieving adequate postoperative weight loss, establish realistic expectations, and reinforce the importance of sustained adherence to long‐term weight‐management recommendations [41]. For patients with a high predicted risk of SCR, alternative procedures associated with greater average weight loss, such as Roux‐en‐Y gastric bypass (RYGB) or one‐anastomosis gastric bypass (OAGB) [42, 43], could be considered when clinically appropriate. In addition, the risk estimate may guide proactive and individualized postoperative management rather than the current standard of care, which often delays response until SCR has already occurred—thereby increasing the risk of metabolic relapse and revisional surgery [13, 44]. High‐risk patients may benefit from enhanced postoperative strategies, including intensive weight monitoring [45], individualized nutritional and physical activity regimens [46, 47]. They may also represent a potential target population for early adjunctive pharmacotherapy, such as GLP‐1 receptor agonists [48, 49], which may further enhance weight loss and reduce the likelihood of SCR.

This study has several limitations. First, it was based on a retrospective observational cohort, and thus, selection bias is inevitable. Second, the model was developed and validated exclusively in Chinese patients with obesity undergoing SG; therefore, its generalizability to other ethnic populations and bariatric procedures remains uncertain. Third, the XGB model was developed specifically to predict SCR after SG, an early suboptimal postoperative outcome, and should not be extrapolated to long‐term outcomes such as weight regain, which differs from SCR in its temporal profile, underlying mechanisms, and therapeutic implications [50]. Fourth, psychological factors were not included in the analysis, although previous studies have suggested that they may influence weight loss outcomes [51]. Fifth, although postoperative nutritional strategies were implemented in accordance with American Society for Metabolic and Bariatric Surgery (ASMBS) guidelines [28, 52] across all participating centers, patient adherence to these managements was not assessed, which should be considered when interpreting our findings.

## 4 Conclusion

We developed and externally validated a machine‐learning model for predicting SCR after SG. The XGB model outperformed clinical experts and may enable early identification of patients at high risk for poor weight loss outcomes. Its application in preoperative risk stratification may help guide timely intervention, optimize personalized management, and support more precise clinical decision‐making in bariatric surgery.

## 5 Materials and Methods

This study was performed in accordance with the Helsinki Declaration and was approved by the Institutional Review Board of all the involved hospitals (2023‐KY‐331) on November 29, 2023. The requirement for informed consent was waived since this was an observational and retrospective study. The TRIPOD (Transparent Reporting of a multivariable prediction model for Individual Prognosis Or Diagnosis) +AI was used as the reporting guide for our study [53].

### 5.1 Study Design and Participants

The overall study workflow is presented in Figure 1. This multicenter cohort study included consecutive patients with obesity who underwent SG across three tertiary hospitals in China. Patients were stratified into a derivation cohort and two independent external validation cohorts. The derivation cohort comprised patients from China‐Japan Friendship Hospital (May 2017 to February 2022, Beijing, China), while the external validation cohorts included patients from the Third Xiangya Hospital (October 2014 to July 2021, Changsha, Hunan, China; external validation cohort 1) and the Second Hospital of Shandong University (October 2014 to July 2021, Jinan, Shandong, China; external validation cohort 2). We used all eligible patients’ data from three cohorts to maximize power and improve generalizability. The resultant sample size and the number of events exceed all approaches for determining sample sizes and therefore are expected to provide estimates that are very robust.

Patients aged 16–65 years who met the indications for bariatric surgery in the 2022 American Society for Metabolic and Bariatric Surgery and the International Federation for the Surgery of Obesity and Metabolic Disorders guidelines for the Asian obesity population [54], underwent standard SG, and completed 1‐year postoperative follow‐up were included in this study. The exclusion criteria included (1) those who underwent revisional bariatric procedures; (2) female patients who became pregnant within 1 year postoperatively; (3) patients who used anti‐obesity pharmacotherapy within 1 year after surgery; (4) those who underwent other gastrointestinal surgeries within 1 year; and (5) individuals with a history of malignancy.

### 5.2 Surgical Procedure and Dietary Management

A symmetric three‑port approach was applied to perform the standard SG procedure in China‐Japan Friendship Hospital and the Second Hospital of Shandong University. The lateral omentum of the greater curvature of the stomach was divided using an ultrasonic scalpel starting from the distal antrum (4–6 cm proximal to the pylorus) up to the angle of His. Subsequently, the stomach was vertically transected using multiple laparoscopic linear staplers, running parallel to and alongside a 36‐Fr bougie tube. A similar surgical technique was employed at the Third Xiangya Hospital, utilizing a symmetric five‐port approach and a 32‐Fr bougie tube.

Dietary management was standardized within each participating center according to the clinical practice guidelines of the ASMBS [28, 52].

### 5.3 Data Collection

Before data collection commenced, all investigators involved in data extraction received standardized training to ensure consistency. Clinical and laboratory data were systematically recorded using a standardized electronic spreadsheet. At each participating hospital, two investigators independently extracted the data and cross‐verified the results to ensure accuracy and reliability. In total, 41 clinical and laboratory variables were obtained from electronic medical records.

Among these, 12 were clinical variables, including sex, age, height, weight, body mass index (BMI), waist circumference, hip circumference, waist‐to‐hip ratio, smoking history, alcohol consumption, hypertension, and T2DM. Anthropometric measurements (height, weight, waist and hip circumferences) were obtained on the morning of surgery with patients in a fasting state, barefoot, and wearing minimal clothing, following the World Health Organization guidelines [55].

Hypertension was defined as a systolic blood pressure ≥ 140 mmHg, a diastolic blood pressure ≥ 90 mmHg, or a prior diagnosis of hypertension [56]. T2DM was diagnosed according to the American Diabetes Association criteria [57], which included the following: FPG ≥ 7.0 mmol/L, 2‐h plasma glucose ≥ 11.1 mmol/L during an oral glucose tolerance test, glycated hemoglobin (HbA1c) ≥ 6.5%, the presence of classic symptoms of hyperglycemia or hyperglycemic crisis with a random plasma glucose ≥ 11.1 mmol/L, or a previous clinical diagnosis of T2DM.

All laboratory tests were performed using fasting blood samples collected within 14 days before surgery. Given the inter‐hospital variability in measurement units, all laboratory values were converted to standardized units prior to analysis. The 29 laboratory variables collected included FPG, fasting insulin, fasting C‐peptide, HbA1c, alanine aminotransferase, aspartate aminotransferase, GGT, uric acid, creatinine, TC, triglycerides, high‐density lipoprotein cholesterol, low‐density lipoprotein cholesterol, lipoprotein (a), calcium, thyroid‐stimulating hormone, free triiodothyronine, free thyroxine, total triiodothyronine, total thyroxine, hemoglobin, folate, vitamin B12, ferritin, estradiol, testosterone, follicle stimulating hormone, luteinizing hormone, and prolactin. Reference ranges and assay methods for these laboratory variables are detailed in Table S6.

Variables with more than 15% missing data were excluded before model development to reduce imputation‐related bias. Missing values were handled using multiple imputation by chained equations with the “mice” package in R [58]. After imputation, the completed dataset was used for subsequent model training and validation. Table S7 summarizes the comparison between the datasets before and after imputation, with no statistical differences observed.

### 5.4 Outcome Variable

Weight loss outcomes were measured by the %TWL, which was calculated as $100\%\times \frac{\mathrm{baseline}\;\mathrm{weight}\;-\;\mathrm{postoperative}\;\mathrm{weight}}{\mathrm{baseline}\;\mathrm{weight}}$. The outcome variable was the occurrence of SCR at 1 year following SG. According to the findings from previous literature, SCR was defined as %TWL < 25%, while OCR was defined as %TWL ≥ 25% [19, 22, 25].

### 5.5 Model Development

The derivation cohort was randomly divided into the training set and testing set in a 4:1 ratio using stratified sampling. Continuous variables were standardized to have a mean of zero and a standard deviation of one. Six ML algorithms were implemented: logistic regression [59], naive Bayes [60], neural network [61], quadratic discriminant analysis [62], support vector machine [63], and XGB [27].

Hyperparameter tuning was conducted for all candidate algorithms within the training set using grid search combined with fivefold cross‐validation, based on all 41 candidate variables as input features. For each candidate hyperparameter combination, model performance was evaluated using the mean cross‐validated AUC. The combination yielding the highest mean AUC was selected as the optimal configuration. The final tuned model was then refitted on the entire training set and evaluated in the internal testing set. The final optimal hyperparameter settings for all algorithms are provided in Table S8. Among these algorithms, the XGB model demonstrated the best AUC, sensitivity, NPV, and F1 score and was therefore selected for further analysis (Figure S7).

To identify the smallest predictor subset that retained model performance, the 41 candidate variables were ranked in descending order by their absolute SHAP values (Figure S8) and sequentially incorporated into the XGB model. Model discrimination was reassessed after the addition of each variable, and the subset preceding the point at which the AUC ceased to improve was retained for further analysis. The AUC plateaued and subsequently decreased slightly after the 12th variable was added (Figure S9A). Accordingly, the top 11 predictors were initially selected: age, BMI, FPG, HbA1c, T2DM, weight, hip circumference, vitamin B12, calcium, TC, and GGT. Pearson correlation analysis was subsequently performed to identify and remove highly correlated predictors. As shown in Figure S9B, strong correlations were observed between FPG and HbA1c and among BMI, weight and hip circumference. Within each correlated group, FPG and BMI were retained because they had higher SHAP values than the corresponding correlated variables. The final XGB model therefore included eight predictors: BMI, age, FPG, T2DM, vitamin B12, calcium, TC, and GGT.

All ML procedures were implemented using Python (version 3.9.9), NumPy (version 1.23.5), and scikit‐learn (version 1.2.2).

### 5.6 Model Evaluation

ROC curve analysis was used to assess model discrimination, with AUC values reported alongside bias‐corrected 95% CIs, calculated by bootstrapping with 1000 resamples [64]. The optimal cut‐off value was determined by maximizing the Youden index, and corresponding evaluation metrics including accuracy, sensitivity, specificity, positive predictive value (PPV), negative predictive value (NPV), and F1 score were subsequently calculated. Calibration performance was assessed using the Brier score, which measures the mean squared difference between predicted probabilities and actual outcomes. Lower Brier scores indicate better agreement between predicted risk and observed events [65]. Clinical utility was assessed through DCA [66]. The 95% CIs for the evaluation metrics were obtained through 1000 bootstrap resamples. Subgroup analyses were conducted by stratifying the population based on clinical characteristics, including sex (male or female), age (< 40 years or ≥ 40 years), BMI (< 37.5 kg/m^2^ or ≥ 37.5 kg/m^2^), T2DM (presence or absence), hypertension (presence or absence), and bougie tube size (36‐Fr or 32‐Fr).

### 5.7 Comparison With Human Experts

Ten clinical experts with varying levels of experience from four independent centers participated in the expert prediction task (Table S9). Based on their clinical experience, the experts were categorized into three groups: junior (≤ 5 years), middle‐level (6–10 years), and senior (> 10 years). All experts were blinded to identifiable information of patients and postoperative outcomes, and none were involved in model development or patient care. Each expert was given access to the full set of 41 candidate variables and independently evaluated every patient in the external validation cohort. For each case, the experts were asked to provide two judgments: (1) whether the patient would develop SCR at 1 year after SG, and (2) whether the case was considered challenging to predict.

The predictive performance of the experts was directly compared with that of the XGB model. The AUC values were compared using 1000 bootstrap resamples [64]. In addition, the categorical NRI was used to evaluate the extent to which the model improved classification accuracy relative to expert judgment [67]. The consistency among expert predictions was assessed using the ICC [68].

Additionally, patients whose outcomes were considered challenging to predict by five or more experts were classified as “challenging cases.” The predictive performance of the XGB model was subsequently evaluated in this subgroup and compared with that of the clinical experts to examine whether the model could improve prediction reliability in these difficult cases.

### 5.8 Model Interpretation

To enhance model interpretability, the SHAP method was applied to rank the importance of input features, provide insights into model behavior and visualize the contribution of individual features to specific predictions [69].

### 5.9 Statistical Analysis

Patient characteristics were summarized as either continuous or categorical variables. For continuous variables with a normal distribution, data were expressed as mean ± standard deviation and compared using the Student's *t* test. For non‐normally distributed continuous variables, the Mann–Whitney *U* test was used, and results were presented as median [interquartile range]. Categorical variables were reported as counts (percentages), and comparisons were made using the chi‐square test or Fisher exact test, as appropriate. All statistical tests were two‐sided, and *p* values < 0.05 were considered statistically significant. Statistical analyses were conducted using STATA/MP (version 16.0) and R software (version 3.6.3).

## Author Contributions

Yuntao Nie, Hua Meng, Shaihong Zhu, and Yinlu Ding had full access to and verified all the data in the study and take responsibility for the integrity of the data and the accuracy of the data analysis. Yuntao Nie, Hua Meng, and An Ping conceived of and designed the study. Yuntao Nie and An Ping drafted the article, developed the models, and performed the statistical analyses. An Ping, Tianshi Wang, Baoyin Liu, Biao Zhou, Haibo Tang, Lei Zhang, Ming Zhu, Nianrong Zhang, and Ziru Tian collected the data. Chunhui Li, Genzheng Liu, Jingwei Hu, Pengpeng Wang, Shuman Ran, Siqi Wang, Ximing Lin, Yan Liu, Yishan Huang, and Zhe Wang estimated the SCR risk in blinded condition. Hua Meng, Shaihong Zhu, and Yinlu Ding provided supervision. Hua Meng obtained funding and had the final responsibility for the decision to submit for publication. All authors have read and approved the final manuscript.

## Funding

This research was funded by the Capital's Funds for Health Improvement and Research, grant number 2026‐2‐4063; China‐Japan Friendship Hospital National High Level Hospital Clinical Research Funding, grant number 2025‐NHLHCRF‐DLYJ‐RCT‐05; China‐Japan Friendship Hospital Talent Introduction Project, grant number 2018‐RC‐1; Beijing Clinical Research Program for the Construction of Research‐Oriented Wards, grant number 2022‐YJXBF‐03‐02; National Natural Science Foundation of China, grant number 82470857; and Beijing Natural Science Foundation, grant number 7242125. The study funders were not involved in the design of the study; the collection, analysis, and interpretation of data; writing the report; and did not impose any restrictions regarding the publication of the report.

## Ethics Statement

This study was performed in accordance with the Helsinki Declaration and was approved by the Institutional Review Board of all the involved hospitals (2023‐KY‐331). The requirement for informed consent was waived since this was a retrospective study.

## Conflicts of Interest

The authors declare no conflicts of interest.

## Supporting information




**Supporting File**: mco271110‐sup‐0001‐SuppMat.pdf.

## Data Availability

All analytic code is openly available at https://github.com/An‐Ping‐bjmu/Prediction‐model‐for‐SCR. The datasets generated and analyzed during the current study are not publicly available due to the requirements of applicable laws but are available from the corresponding author on reasonable request.
