# Supplementary material for: Development and Validation of an Interpretable Machine‐Learning Model for Predicting Suboptimal Clinical Response After Sleeve Gastrectomy: A Multicenter Study
Source: MedComm (2020). 2026 Nov 3;7(11):e71110. doi: 10.1002/mco2.71110 (PMC13732101; doi:10.1002/mco2.71110)
Supplement: Supplementary file 1 [file MCO2-7-e71110-s001.pdf]

Development and validation of an interpretable machine-learning model for predicting suboptimal clinical response after sleeve gastrectomy: a multicenter study

Running title:

Predicting SCR after sleeve gastrectomy

Authors:

Yuntao Nie[1#], An Ping[2,3#], Tianshi Wang[4#], Haibo Tang[5], Yishan Huang[1], Zhe Wang[1], Pengpeng Wang[1], Siqi Wang[1], Genzheng Liu[1], Ximing Lin[1], Shuman Ran[1], Yan Liu[6], Ming Zhu[7], Jingwei Hu[8], Chunhui Li[9], Nianrong Zhang[1], Baoyin Liu[1], Lei Zhang[10], Biao Zhou[1], Ziru Tian[11], Shaihong Zhu[5*], Yinlu Ding[4*], Hua Meng[1*]

Affiliations:

[1] Department of General Surgery & Obesity and Metabolic Disease Center, China-Japan Friendship Hospital, Beijing, China

[2] School of Basic Medical Sciences, Capital Medical University, Beijing, China

[3] Department of Education, Beijing Luhe Hospital, Capital Medical University, Beijing, China

[4] Department of General Surgery, The Second Hospital of Shandong University, Cheeloo College of Medicine, Shandong University, Jinan, Shandong, China

[5] Department of General Surgery, The Third Xiangya Hospital of Central South University, Changsha, Hunan, China

[6] Department of Gastrointestinal Metabolic Surgery, Zhengzhou Central Hospital, Zhengzhou University, Zhengzhou, Henan, China.

[7] Department of Gastrointestinal Surgery, Jining No. 1 People's Hospital, Jining, Shandong, China.

[8] Department of Gastrointestinal Surgery, The Second Affiliated Hospital of Wenzhou Medical University, Wenzhou, Zhejiang, China

[9] Department of Endocrinology, The Second Hospital of Hebei Medical University, Shijiazhuang, Hebei, China

[10] Department of oncology, Sinopharm Tongmei General Hospital, Datong, Shanxi, China

[11] National Cancer Center/ National Clinical Research Center for Cancer/ Cancer Hospital, Chinese Academy of Medical Science and Peking Union Medical College, Beijing, China

[#] Yuntao Nie, An Ping, and Tianshi Wang contributed equally to this work.

***Corresponding authors:**

Hua Meng (lead contact), MD, Department of General Surgery & Obesity and Metabolic Disease Center, China-Japan Friendship Hospital, 2 East Yinghuayuan Street, Chaoyang District, Beijing, 100029, China. Email: menghua@zryhyy.com.cn

Yinlu Ding, MD, Department of General Surgery, The Second Hospital of Shandong University, Cheeloo College of Medicine, 247 Bei Yuan Street, Shandong University, Shandong Jinan, 250033, China. Email: dingyinlu@126.com

Shaihong Zhu, MD, Department of General Surgery, The Third Xiangya Hospital of Central South University, 138 Tongzipo Street, Changsha Hunan, 410013, China. Email: shaihongzhu@126.com

Table S1. Comparison of characteristics between OCR and SCR groups in different cohort

Characteristics	Derivation cohort (N=1094)						External validation cohort (n=218)		
	Training cohort (n=871)			Testing cohort (n=223)					
	OCR (n=573)	SCR (n=298)	P-value	OCR (n=147)	SCR (n=76)	P-value	OCR (n=139)	SCR (n=79)	P-value
Male, n (%)	187 (32.6)	85 (28.5)	0.244	58 (39.5)	15 (19.7)	0.005	53 (38.1)	15 (19.0)	0.005
Age, years	33.3 ± 8.0	37.2 ± 9.4	<0.001	31.8 ± 8.7	38.1 ± 9.7	<0.001	31.7 ± 7.1	36.0 ± 9.2	<0.001
Height, cm	168.0 [163.0, 175.0]	167.0 [162.0, 173.0]	0.010	169.0 [163.0, 178.0]	165.0 [160.8, 170.0]	<0.001	169.0 [163.8, 174.0]	163.0 [159.5, 168.5]	<0.001
Weight, kg	112.0 [97.0, 131.0]	100.0 [91.0, 113.9]	<0.001	116.0 [100.0, 135.0]	90.0 [83.0, 106.0]	<0.001	110.0 [96.0, 130.0]	92.7 [85.0, 105.3]	<0.001
BMI, kg/m²	39.3 [35.3, 44.6]	35.8 [32.5, 39.8]	<0.001	40.1 [36.3, 45.6]	33.2 [31.4, 36.7]	<0.001	38.7 [34.0, 44.6]	34.6 [31.9, 38.0]	<0.001
Waist circumference, cm	119.0 [110.0, 132.0]	112.0 [105.0, 122.0]	<0.001	120.0 [110.0, 131.0]	107.0 [100.8, 117.3]	<0.001	119.0 [108.0, 133.5]	108.0 [101.0, 116.0]	<0.001
Hip circumference, cm	125.0 [116.0, 133.0]	117.0 [110.0, 126.0]	<0.001	126.0 [118.0, 137.0]	113.0 [107.0, 119.3]	<0.001	124.0 [115.0, 136.0]	112.0 [106.0, 120.5]	<0.001
Waist-to-hip ratio	1.0 ± 0.1	1.0 ± 0.1	0.620	1.0 ± 0.1	1.0 ± 0.1	0.761	1.0 ± 0.1	1.0 ± 0.1	0.939
Smoking history, n (%)	113 (19.7)	43 (14.4)	0.066	25 (17.0)	16 (21.1)	0.578	22 (15.8)	6 (7.6)	0.125
Alcohol consumption, n (%)	68 (11.9)	29 (9.7)	0.403	14 (9.5)	5 (6.6)	0.622	12 (8.6)	5 (6.3)	0.729
Hypertension, n (%)	219 (38.2)	135 (45.3)	0.052	59 (40.1)	24 (31.6)	0.268	51 (36.7)	31 (39.2)	0.82
T2DM, n (%)	246 (42.9)	186 (62.4)	<0.001	51 (34.7)	50 (65.8)	<0.001	39 (28.1)	40 (50.6)	0.001
FPG, mmol/L	5.7 [5.1, 6.8]	6.4 [5.3, 8.2]	<0.001	5.5 [4.8, 6.3]	6.7 [5.4, 8.7]	<0.001	5.3 [4.8, 6.06]	5.9 [5.1, 8.5]	<0.001
Ins, μIU/ml	28.5 ± 18.1	25.8 ± 21.9	0.051	28.7 ± 21.6	21.0 ± 10.3	0.004	31.4 ± 18.4	27.5 ± 20.1	0.143
C-P, ng/ml	4.6 ± 3.3	4.2 ± 2.8	0.102	4.4 ± 2.3	3.7 ± 1.2	0.015	4.4 ± 1.8	4.1 ± 1.8	0.227
HbA1c, %	5.9 [5.5, 6.7]	6.3 [5.6, 7.7]	<0.001	5.8 [5.4, 6.3]	6.6 [5.6, 8.6]	<0.001	5.9 [5.5, 6.3]	6.0 [5.6, 7.3]	0.045
ALT, IU/L	40.0 [26.0, 70.0]	35.0 [23.0, 61.0]	0.038	37.0 [24.5, 56.5]	38.5 [21.0, 58.8]	0.483	35.0 [18.9, 78.0]	29.0 [20.1, 42.0]	0.248
AST, IU/L	24.0 [17.0, 38.0]	24.0 [17.3, 36.0]	0.790	23.0 [18.0, 32.5]	23.0 [17.0, 35.0]	0.863	27.4 [19.0, 41.1]	25.0 [19.0, 40.5]	0.713
GGT, IU/L	45.7 ± 44.0	40.2 ± 29.2	0.052	45.2 ± 33.4	40.1 ± 38.2	0.310	48.4 ± 35.5	48.0 ± 30.9	0.934
UA, μmol/L	451.4 ± 221.0	441.3 ± 231.8	0.527	439.4 ± 109.4	380.9 ± 97.2	<0.001	407.2 ± 91.7	392.6 ± 105.0	0.287
TC, mmol/L	4.9 ± 1.0	4.8 ± 1.0	0.802	4.9 ± 0.9	4.8 ± 0.9	0.421	5.0 ± 0.9	5.0 ± 1.2	0.841
Triglyceride, mmol/L	2.2 ± 2.4	2.3 ± 1.6	0.608	2.1 ± 1.6	2.1 ± 1.0	0.762	2.0 ± 1.6	2.5 ± 3.1	0.076
HDL-C, mmol/L	1.0 ± 0.2	1.0 ± 0.2	0.358	1.0 ± 0.2	1.0 ± 0.2	0.801	1.2 ± 0.3	1.1 ± 0.3	0.668
LDL-C, mmol/L	3.0 ± 0.7	3.0 ± 0.8	0.711	3.1 ± 0.7	3.0 ± 0.7	0.274	3.0 ± 0.7	2.8 ± 0.9	0.092
LP(a), mg/L	63.4 [33.0, 130.4]	70.8 [35.3, 151.4]	0.238	76.5 [40.5, 154.4]	64.9 [38.1, 174.1]	0.726	158.0 [80.0, 494.0]	111.3 [52.0, 388.5]	0.072
Ca, mmol/L	2.3 ± 0.1	2.3 ± 0.1	0.040	2.3 ± 0.2	2.3 ± 0.1	0.926	2.3 ± 0.1	2.3 ± 0.2	0.074
Creatinine, μmol/L	60.2 ± 22.0	60.1 ± 26.7	0.961	62.6 ± 20.8	55.5 ± 18.0	0.011	61.7 ± 13.1	57.1 ± 18.4	0.033
TSH, μIU/L	2.9 ± 3.1	2.6 ± 1.9	0.131	3.0 ± 2.3	2.7 ± 1.9	0.365	2.4 ± 1.4	2.3 ± 1.5	0.654

FT3, pg/ml	3.3 ± 0.5	3.2 ± 0.5	0.009	3.3 ± 0.4	3.2 ± 0.4	0.026	3.7 ± 0.6	3.8 ± 1.6	0.731
FT4, ng/dl	1.1 ± 0.3	1.1 ± 0.3	0.640	1.1 ± 0.2	1.1 ± 0.3	0.685	1.0 ± 0.3	1.1 ± 0.6	0.025
TT3, ng/ml	1.2 ± 0.2	1.1 ± 0.2	0.121	1.2 ± 0.2	1.1 ± 0.2	0.051	1.3 ± 0.3	1.3 ± 0.4	0.384
TT4, μg/dl	8.3 ± 1.8	8.3 ± 1.9	0.997	8.5 ± 2.0	8.3 ± 1.7	0.531	8.7 ± 3.1	9.1 ± 3.3	0.363
Hemoglobin, g/L	136.7 ± 18.1	136.1 ± 16.2	0.636	139.3 ± 15.8	133.1 ± 20.8	0.013	143.6 ± 15.7	137.5 ± 17.1	0.009
Folate, nmol/L	17.9 [12.7, 27.8]	21.3 [14.2, 33.2]	<0.001	17.3 [12.1, 24.4]	22.7 [14.2, 42.0]	0.001	9.0 [7.0, 14.3]	8.8 [6.4, 13.2]	0.719
Vitamin B12, pmol/L	215.8 [168.0, 288.7]	230.0 [168.0, 320.8]	0.059	215.0 [165.7, 277.2]	242.0 [173.0, 293.8]	0.191	294.9 [238.5, 400.5]	327.0 [248.2, 572.7]	0.012
Ferritin, ng/ml	80.2 [39.4, 167.4]	76.3 [37.5, 156.0]	0.748	88.7 [45.8, 178.0]	73.4 [43.4, 109.0]	0.075	160.0 [72.7, 293.8]	89.5 [43.3, 178.0]	0.006
Estradiol, pmol/L	215.1 [137.2, 521.5]	256.1 [130.1, 560.2]	0.985	203.0 [137.4, 544.6]	174.6 [105.0, 435.2]	0.078	130.5 [102.0, 216.2]	141.0 [99.6, 277.2]	0.64
Testosterone, nmol/L	4.1 [2.0, 8.6]	3.7 [1.7, 8.2]	0.206	5.1 [2.0, 9.5]	2.1 [1.3, 5.2]	<0.001	2.4 [1.7, 6.5]	1.7 [1.1, 2.6]	<0.001
FSH, mIU/ml	4.8 [3.4, 6.6]	5.5 [3.6, 7.7]	0.004	4.9 [3.2, 6.5]	6.0 [4.1, 8.3]	0.001	4.4 [2.9, 6.1]	5.4 [3.9, 7.3]	0.011
LH, mIU/ml	5.1 [3.3, 7.5]	5.5 [3.3, 8.9]	0.103	4.8 [3.1, 7.5]	5.1 [3.0, 9.0]	0.469	6.0 [3.5, 8.9]	5.5 [3.5, 8.5]	0.486
Prolactin, mIU/L	425.5 [261.3, 4472.1]	440.1 [270.4, 4570.6]	0.651	455.0 [259.2, 4244.8]	385.6 [228.3, 3156.7]	0.196	273.4 [197.9, 368.2]	209.8 [153.7, 284.8]	0.003

The categorical variables of the cohort data were expressed as n (%), and continuous variables with normal distribution were expressed as mean ± standard deviation, while medians [quartiles] were used for variables that did not satisfy normal distribution.

The p-values refer to the comparison between OCR and SCR groups with the use of Student t test or the Mann–Whitney U test for continuous variables and Fisher exact test or Pearson x^2 test.

Abbreviations, ALT; alanine transpeptidase; AST, aspartate transaminase; BMI, body mass index; Ins, fasting insulin; Ca, serum calcium; C-P, fasting C-peptide; FPG, fasting plasma glucose; FSH, follicle stimulating hormone; FT3, free triiodothyronine; FT4, free thyroxine; GGT, gamma glutamyl transferase; HbA1c, glycated hemoglobin; HDL-C, high-density lipoprotein cholesterol; Ins, fasting insulin; LDL-C, low-density lipoprotein cholesterol; LH, luteinizing Hormone; LP(a), lipoprotein (a); OCR, optimal clinical response; SCR, suboptimal clinical response; TC, total cholesterol; TSH, thyroid-stimulating hormone; TT3, total triiodothyronine; TT4, total thyroxine; T2DM, type 2 diabetes mellitus; UA, uric acid.

Table S2. Performance of the XGB model in different subgroups

Subgroup group	n	AUC (95% CI)	Accuracy (95% CI)	Sensitivity (95% CI)	Specificity (95% CI)	PPV (95% CI)	NPV (95% CI)	F1 score (95% CI)	Brier score (95% CI)
**Testing set (n=223)**									
Age ≥ 40 years	48	0.866 (0.721-0.946)	0.646 (0.500-0.771)	1.000	0.150 (0.000-0.368)	0.622 (0.468-0.756)	1.000	0.767 (0.638-0.866)	0.188 (0.166-0.214)
Age < 40 years	175	0.797 (0.703-0.860)	0.771 (0.703-0.828)	0.688 (0.544-0.800)	0.803 (0.725-0.867)	0.569 (0.442-0.695)	0.872 (0.803-0.925)	0.623 (0.516-0.727)	0.166 (0.143-0.192)
Male	73	0.779 (0.580-0.892)	0.795 (0.685-0.863)	0.867 (0.559-1.000)	0.776 (0.643-0.860)	0.500 (0.284-0.684)	0.957 (0.851-1.000)	0.634 (0.437-0.809)	0.140 (0.110-0.176)
Female	150	0.817 (0.735-0.881)	0.720 (0.627-0.780)	0.787 (0.667-0.875)	0.674 (0.571-0.770)	0.623 (0.509-0.726)	0.822 (0.716-0.896)	0.696 (0.604-0.770)	0.186 (0.161-0.215)
BMI ≥ 37.5 Kg/m2	117	0.706 (0.520-0.828)	0.778 (0.684-0.838)	0.500 (0.200-0.714)	0.822 (0.739-0.891)	0.308 (0.150-0.500)	0.912 (0.829-0.957)	0.381 (0.205-0.561)	0.125 (0.100-0.157)
BMI < 37.5 Kg/m2	106	0.765 (0.660-0.846)	0.708 (0.604-0.783)	0.883 (0.787-0.948)	0.478 (0.334-0.615)	0.688 (0.567-0.775)	0.759 (0.520-0.875)	0.774 (0.674-0.839)	0.221 (0.196-0.250)
T2DM	101	0.808 (0.706-0.879)	0.703 (0.594-0.772)	0.940 (0.830-0.981)	0.471 (0.333-0.611)	0.635 (0.514-0.743)	0.889 (0.701-0.967)	0.758 (0.672-0.836)	0.195 (0.172-0.224)
Non-T2DM	122	0.762 (0.635-0.856)	0.779 (0.680-0.836)	0.538 (0.333-0.727)	0.844 (0.763-0.913)	0.483 (0.304-0.667)	0.871 (0.781-0.927)	0.509 (0.341-0.667)	0.151 (0.123-0.184)
Hypertension	83	0.831 (0.711-0.911)	0.687 (0.578-0.771)	0.875 (0.672-0.962)	0.610 (0.457-0.721)	0.477 (0.341-0.630)	0.923 (0.787-0.977)	0.618 (0.476-0.757)	0.161 (0.135-0.195)
Non-hypertension	140	0.834 (0.743-0.892)	0.779 (0.693-0.836)	0.769 (0.636-0.871)	0.784 (0.692-0.863)	0.678 (0.536-0.778)	0.852 (0.762-0.920)	0.721 (0.627-0.807)	0.177 (0.151-0.205)
**External validation cohort (n=218)**									
Age ≥ 40 years	42	0.674 (0.431-0.841)	0.643 (0.452-0.762)	0.958 (0.774-1.000)	0.222 (0.056-0.438)	0.622 (0.447-0.765)	0.800 (0.000-1.000)	0.754 (0.615-0.857)	0.227 (0.188-0.269)
Age < 40 years	176	0.744 (0.647-0.820)	0.699 (0.619-0.756)	0.709 (0.583-0.830)	0.694 (0.611-0.773)	0.513 (0.411-0.627)	0.840 (0.762-0.900)	0.595 (0.484-0.688)	0.184 (0.161-0.214)
Male	68	0.790 (0.556-0.913)	0.765 (0.632-0.853)	0.733 (0.444-0.929)	0.774 (0.654-0.870)	0.478 (0.247-0.680)	0.911 (0.773-0.976)	0.579 (0.370-0.756)	0.139 (0.112-0.187)
Female	150	0.731 (0.638-0.811)	0.653 (0.567-0.720)	0.797 (0.680-0.882)	0.547 (0.436-0.656)	0.567 (0.463-0.667)	0.783 (0.658-0.873)	0.662 (0.570-0.745)	0.216 (0.190-0.245)
BMI ≥ 37.5 Kg/m2	98	0.672 (0.485-0.806)	0.776 (0.673-0.847)	0.476 (0.250-0.684)	0.857 (0.760-0.923)	0.476 (0.263-0.696)	0.857 (0.756-0.922)	0.476 (0.279-0.654)	0.160 (0.128-0.202)
BMI < 37.5 Kg/m2	120	0.737 (0.628-0.820)	0.617 (0.517-0.692)	0.897 (0.796-0.957)	0.355 (0.239-0.483)	0.565 (0.467-0.663)	0.786 (0.586-0.909)	0.693 (0.599-0.767)	0.218 (0.194-0.245)
T2DM	79	0.776 (0.652-0.866)	0.709 (0.571-0.785)	0.925 (0.814-0.979)	0.487 (0.333-0.645)	0.649 (0.516-0.768)	0.864 (0.632-1.000)	0.763 (0.659-0.854)	0.209 (0.180-0.245)
Non-T2DM	139	0.694 (0.579-0.793)	0.676 (0.590-0.741)	0.641 (0.479-0.780)	0.690 (0.598-0.774)	0.446 (0.329-0.585)	0.831 (0.744-0.906)	0.526 (0.385-0.634)	0.182 (0.155-0.214)
Hypertension	82	0.813 (0.699-0.892)	0.732 (0.602-0.805)	0.839 (0.667-0.939)	0.667 (0.532-0.787)	0.605 (0.438-0.737)	0.872 (0.727-0.951)	0.703 (0.563-0.805)	0.177 (0.146-0.213)
Non-hypertension	136	0.729 (0.620-0.805)	0.662 (0.574-0.735)	0.750 (0.609-0.854)	0.614 (0.506-0.722)	0.514 (0.403-0.627)	0.818 (0.709-0.899)	0.610 (0.501-0.711)	0.202 (0.176-0.236)
32-Fr bougie tube	84	0.770 (0.648-0.862)	0.714 (0.606-0.798)	0.765 (0.597-0.879)	0.680 (0.549-0.794)	0.619 (0.463-0.750)	0.810 (0.651-0.900)	0.684 (0.541-0.795)	0.193 (0.164-0.232)
36-Fr bougie tube	134	0.751 (0.659-0.833)	0.672 (0.575-0.739)	0.800 (0.652-0.905)	0.607 (0.494-0.707)	0.507 (0.386-0.615)	0.857 (0.750-0.924)	0.621 (0.510-0.721)	0.191 (0.167-0.222)

Abbreviations: AUC, area under the receiver operating characteristic curve; BMI, body mass index; NPV, negative predictive value; PPV, positive predictive value; T2DM, type 2 diabetes mellitus.

Table S3. Performance of the XGB model and its comparison with human experts in the external validation cohort 1

Model and experts	Years of experience	AUC Observation (95% CI)	AUC P-value	Accuracy (95% CI)	Sensitivity (95% CI)	Specificity (95% CI)	PPV (95% CI)	NPV (95% CI)	F1 score (95% CI)	Brier score (95% CI)
XGB model	NA	0.770 (0.648-0.862)	Reference	0.714 (0.606-0.798)	0.765 (0.597-0.879)	0.680 (0.549-0.794)	0.619 (0.463-0.750)	0.810 (0.651-0.900)	0.684 (0.541-0.795)	0.193 (0.164-0.232)
Average Junior experts	<5	0.511 (0.473-0.559)	0.003	0.524 (0.484-0.568)	0.441 (0.378-0.506)	0.580 (0.532-0.642)	0.490 (0.340-0.629)	0.634 (0.446-0.769)	0.369 (0.299-0.461)	0.476 (0.425-0.512)
Junior expert 1	2	0.568 (0.504-0.657)	<0.001	0.643 (0.512-0.726)	0.176 (0.066-0.344)	0.960 (0.850-1.000)	0.750 (0.250-1.000)	0.632 (0.513-0.729)	0.286 (0.129-0.489)	0.357 (0.250-0.476)
Junior expert 2	4	0.443 (0.355-0.535)	<0.001	0.488 (0.381-0.595)	0.206 (0.094-0.390)	0.680 (0.522-0.796)	0.304 (0.143-0.500)	0.557 (0.435-0.672)	0.246 (0.115-0.407)	0.512 (0.381-0.607)
Junior expert 3	4	0.521 (0.468-0.578)	<0.001	0.440 (0.321-0.536)	0.941 (0.791-1.000)	0.100 (0.038-0.205)	0.416 (0.310-0.519)	0.714 (0.210-1.000)	0.577 (0.466-0.685)	0.560 (0.440-0.655)
Average middle-level experts	6-10	0.552 (0.489-0.622)	0.005	0.603 (0.508-671)	0.284 (0.173-0.412)	0.820 (0.733-0.876)	0.556 (0.298-0.729)	0.629 (0.498-0.720)	0.349 (0.237-0.492)	0.397 (0.318-0.484)
Middle-level expert 1	7	0.497 (0.396-0.604)	<0.001	0.536 (0.417-0.631)	0.294 (0.153-0.459)	0.700 (0.553-0.818)	0.400 (0.222-0.591)	0.593 (0.453-0.714)	0.339 (0.204-0.509)	0.464 (0.357-0.548)
Middle-level expert 2	7	0.621 (0.518-0.726)	0.016	0.655 (0.541-0.738)	0.441 (0.290-0.621)	0.800 (0.649-0.896)	0.600 (0.391-0.798)	0.678 (0.551-0.800)	0.508 (0.353-0.667)	0.345 (0.238-0.440)
Middle-level expert 3	7	0.539 (0.486-0.618)	<0.001	0.619 (0.500-0.702)	0.118 (0.030-0.257)	0.960 (0.861-1.000)	0.667 (0.000-1.000)	0.615 (0.500-0.726)	0.200 (0.053-0.392)	0.381 (0.274-0.476)
Average senior experts	>10	0.503 (0.457-0.548)	<0.001	0.554 (0.471-0.613)	0.235 (0.159-0.309)	0.770 (0.714-0.826)	0.424 (0.288-0.571)	0.597 (0.474-0.689)	0.295 (0.212-0.381)	0.446 (0.382-0.524)
Senior expert 1	12	0.592 (0.517-0.679)	0.002	0.655 (0.536-0.738)	0.265 (0.130-0.440)	0.920 (0.804-0.980)	0.692 (0.364-0.909)	0.648 (0.522-0.745)	0.383 (0.216-0.571)	0.345 (0.238-0.440)
Senior expert 2	13	0.444 (0.364-0.531)	<0.001	0.500 (0.393-0.596)	0.147 (0.050-0.289)	0.740 (0.600-0.844)	0.278 (0.086-0.518)	0.561 (0.439-0.672)	0.192 (0.075-0.360)	0.500 (0.381-0.595)
Senior expert 3	15	0.531 (0.429-0.638)	0.001	0.560 (0.440-0.643)	0.382 (0.228-0.571)	0.680 (0.544-0.804)	0.448 (0.267-0.650)	0.618 (0.481-0.740)	0.413 (0.262-0.577)	0.440 (0.321-0.536)
Senior expert 4	19	0.444 (0.368-0.543)	<0.001	0.500 (0.381-0.597)	0.147 (0.054-0.311)	0.740 (0.600-0.854)	0.278 (0.118-0.532)	0.561 (0.442-0.677)	0.192 (0.078-0.382)	0.500 (0.393-0.595)

The p-values refer to the comparison of the AUC between XGB model and experts through bootstrapping with 1,000 resamples.

Abbreviations: AUC, area under the receiver operating characteristic curve; NA, not applicable; NPV, negative predictive value; PPV, positive predictive value.

Table S4. Performance of the XGB model and its comparison with human experts in the external validation cohort 2

Model and experts	Years of experience	AUC		Accuracy (95% CI)	Sensitivity (95% CI)	Specificity (95% CI)	PPV (95% CI)	NPV (95% CI)	F1 score (95% CI)	Brier score (95% CI)
		Observation (95% CI)	P-value							
XGB model	NA	0.751 (0.659-0.833)	Reference	0.672 (0.575-0.739)	0.800 (0.652-0.905)	0.607 (0.494-0.707)	0.507 (0.386-0.615)	0.857 (0.750-0.924)	0.621 (0.510-0.721)	0.191 (0.167-0.222)
Average Junior experts	<5	0.461 (0.379-0.517)	<0.001	0.493(0.445-0.543)	0.363 (0.287-0.442)	0.558 (0.498-0.615)	0.296 (0.173-0.486)	0.628 (0.529-0.733)	0.273 (0.210-0.357)	0.507 (0.450-0.550)
Junior expert 1	2	0.500 (0.462-0.554)	<0.001	0.642 (0.552-0.709)	0.067 (0.020-0.170)	0.933 (0.859-0.977)	0.333 (0.000-0.667)	0.664 (0.579-0.740)	0.111 (0.036-0.271)	0.358 (0.269-0.440)
Junior expert 2	4	0.397 (0.291-0.492)	<0.001	0.433 (0.343-0.507)	0.289 (0.161-0.436)	0.506 (0.400-0.615)	0.228 (0.127-0.351)	0.584 (0.466-0.695)	0.255 (0.162-0.383)	0.567 (0.470-0.649)
Junior expert 3	4	0.485 (0.400-0.559)	<0.001	0.403 (0.314-0.478)	0.733 (0.592-0.851)	0.236 (0.155-0.349)	0.327 (0.237-0.421)	0.636 (0.461-0.793)	0.452 (0.356-0.557)	0.597 (0.507-0.672)
Average middle-level experts	6-10	0.517 (0.472-0.581)	<0.001	0.609 (0.548-0.672)	0.237 (0.159-0.350)	0.798 (0.749-0.850)	0.378 (0.221-0.607)	0.674 (0.583-0.752)	0.269 (0.193-0.387)	0.391 (0.326-0.448)
Middle-level expert 1	7	0.487 (0.403-0.578)	<0.001	0.545 (0.455-0.612)	0.311 (0.183-0.468)	0.663 (0.554-0.757)	0.318 (0.182-0.449)	0.656 (0.548-0.753)	0.315 (0.193-0.444)	0.455 (0.366-0.544)
Middle-level expert 2	7	0.560 (0.481-0.649)	<0.001	0.634 (0.545-0.709)	0.333 (0.202-0.470)	0.787 (0.688-0.862)	0.441 (0.276-0.600)	0.700 (0.596-0.784)	0.380 (0.258-0.524)	0.366 (0.284-0.440)
Middle-level expert 3	7	0.505 (0.468-0.562)	<0.001	0.649 (0.560-0.716)	0.067 (0.020-0.184)	0.944 (0.874-0.978)	0.375 (0.000-0.800)	0.667 (0.572-0.740)	0.113 (0.036-0.276)	0.351 (0.261-0.425)
Average senior experts	>10	0.491 (0.455-0.536)	<0.001	0.597 (0.528-0.657)	0.167 (0.112-0.245)	0.815 (0.771-0.856)	0.329 (0.185-0.493)	0.659 (0.572-0.737)	0.202 (0.139-0.299)	0.403 (0.339-0.466)
Senior expert 1	12	0.522 (0.485-0.586)	<0.001	0.664 (0.582-0.731)	0.089 (0.025-0.206)	0.955 (0.890-0.988)	0.500 (0.00-0.778)	0.675 (0.595-0.752)	0.151 (0.041-0.312)	0.336 (0.254-0.418)
Senior expert 2	13	0.432 (0.378-0.490)	<0.001	0.552 (0.463-0.633)	0.067 (0.018-0.174)	0.798 (0.706-0.864)	0.143 (0.036-0.355)	0.628 (0.539-0.714)	0.091 (0.028-0.253)	0.448 (0.358-0.530)
Senior expert 3	15	0.515 (0.426-0.608)	<0.001	0.567 (0.470-0.642)	0.356 (0.227-0.510)	0.674 (0.564-0.761)	0.356 (0.227-0.511)	0.674 (0.576-0.768)	0.356 (0.238-0.505)	0.433 (0.342-0.515)
Senior expert 4	19	0.494 (0.434-0.568)	<0.001	0.604 (0.514-0.679)	0.156 (0.063-0.287)	0.831 (0.750-0.902)	0.318 (0.136-0.549)	0.661 (0.569-0.741)	0.209 (0.095-0.350)	0.396 (0.306-0.470)

The p-values refer to the comparison of the AUC between XGB model and experts through bootstrapping with 1,000 resamples.

Abbreviations: AUC, area under the receiver operating characteristic curve; NA, not applicable; NPV, negative predictive value; PPV, positive predictive value.

Table S5. Performance of the XGB model and its comparison with human experts in patients considered challenging by the experts

Model and experts	Years of experience	AUC		Accuracy (95% CI)	Sensitivity (95% CI)	Specificity (95% CI)	PPV (95% CI)	NPV (95% CI)	F1 score (95% CI)	Brier score (95% CI)
		Observation (95% CI)	P-value							
XGB model	NA	0.745 (0.598-0.852)	Reference	0.603 (0.468-0.706)	0.800 (0.567-0.920)	0.488 (0.325-0.639)	0.476 (0.320-0.622)	0.808 (0.592-0.923)	0.597 (0.456-0.734)	0.196 (0.165-0.235)
Average Junior experts	<5	0.452 (0.378-0.497)	<0.001	0.505 (0.427-0.574)	0.253 (0.177-0.321)	0.651 (0.561-0.733)	0.183 (0.109-0.288)	0.600 (0.461-0.722)	0.312 (0.212-0.422)	0.495 (0.417-0.564)
Junior expert 1	2	0.488 (0.432-0.500)	<0.001	0.618 (0.485-0.706)	0.000	0.977 (0.842-1.000)	0.000	0.627 (0.505-0.735)	NA	0.382 (0.25-0.485)
Junior expert 2	4	0.371 (0.278-0.675)	<0.001	0.426 (0.309-0.544)	0.160 (0.042-0.326)	0.581 (0.415-0.732)	0.182 (0.048-0.389)	0.543 (0.381-0.683)	0.170 (0.047-0.347)	0.574 (0.429-0.676)
Junior expert 3	4	0.498 (0.392-0.597)	0.005	0.471 (0.338-0.574)	0.600 (0.375-0.760)	0.395 (0.250-0.545)	0.366 (0.220-0.521)	0.630 (0.440-0.811)	0.455 (0.307-0.603)	0.529 (0.397-0.632)
Average middle-level experts	6-10	0.507 (0.442-0.578)	0.006	0.588 (0.485-0.672)	0.200 (0.107-0.333)	0.814 (0.707-0.880)	0.380 (0.130-0.670)	0.639 (0.504-0.745)	0.231 (0.149-0.354)	0.412 (0.319-0.510)
Middle-level expert 1	7	0.447 (0.373-0.533)	<0.001	0.544 (0.397-0.632)	0.080 (0.000-0.241)	0.814 (0.658-0.902)	0.200 (0.000-0.583)	0.603 (0.446-0.709)	0.114 (0.000-0.286)	0.456 (0.324-0.559)
Middle-level expert 2	7	0.557 (0.425-0.664)	0.017	0.588 (0.446-0.676)	0.440 (0.248-0.640)	0.674 (0.523-0.808)	0.440 (0.241-0.626)	0.674 (0.512-0.800)	0.440 (0.261-0.604)	0.412 (0.273-0.515)
Middle-level expert 3	7	0.517 (0.468-0.602)	0.002	0.632 (0.491-0.735)	0.080 (0.000-0.240)	0.953 (0.848-1.000)	0.500 (0.000-1.000)	0.641 (0.513-0.742)	0.138 (0.000-0.392)	0.368 (0.250-0.471)
Average senior experts	>10	0.496 (0.449-0.540)	0.005	0.596 (0.486-0.683)	0.120 (0.056-0.198)	0.872 (0.804-0.921)	0.363 (0.143-0.562)	0.630 (0.497-0.7310	0.180 (0.098-0.267)	0.404 (0.313-0.507)
Senior expert 1	12	0.533 (0.456-0.627)	0.002	0.632 (0.500-0.721)	0.160 (0.042-0.344)	0.907 (0.793-0.976)	0.500 (0.000-0.800)	0.650 (0.525-0.769)	0.242 (0.074-0.486)	0.368 (0.235-0.471)
Senior expert 2	13	0.513 (0.436-0.593)	0.009	0.618 (0.500-0.735)	0.120 (0.034-0.297)	0.907 (0.778-0.973)	0.429 (0.000-0.857)	0.639 (0.516-0.759)	0.188 (0.057-0.432)	0.382 (0.265-0.485)
Senior expert 3	15	0.459 (0.389-0.540)	<0.001	0.559 (0.426-0.662)	0.080 (0.000-0.258)	0.837 (0.695-0.927)	0.222 (0.000-0.633)	0.610 (0.475-0.732)	0.118 (0.000-0.333)	0.441 (0.324-0.544)
Senior expert 4	19	0.479 (0.400-0.580)	0.001	0.574 (0.441-0.676)	0.120 (0.033-0.300)	0.837 (0.700-0.932)	0.300 (0.000-0.667)	0.621 (0.500-0.738)	0.171 (0.051-0.412)	0.426 (0.294-0.544)

The p-values refer to the comparison of the AUC between XGB model and experts through bootstrapping with 1,000 resamples.

Abbreviations: AUC, area under the receiver operating characteristic curve; NA, not applicable; NPV, negative predictive value; PPV, positive predictive value.

Table S6. The normal ranges and laboratory test method for all laboratory variables

Variables	Normal range	Laboratory test method
FPG	3.61-6.11 mmol/L	Glucose oxidase method
Ins	NA	Radio-immunoassays
C-P	NA	Radio-immunoassays
HbA1c	<6.5%	Liquid chromatography
ALT	0-40 IU/L	Standard enzymatic assay
AST	0-42 IU/L	Standard enzymatic assay
GGT	0-52 IU/L	Standard enzymatic assay
UA	150-420 μmol/L	Standard enzymatic assay
TC	<5.20 mmol/L	Standard enzymatic assay
Triglyceride	<1.70 mmol/L	Standard enzymatic assay
HDL-C	1.00-2.20 mmol/L	Direct method
LDL-C	≤3.4 mmol/L	Direct method
LP(a)	<300 mg/L	Gel clot and kinetic turbidimetric
Calcium	2.00-2.75 mmol/L	Arsenazo III method
Creatinine	35-106 μmol/L	Standard enzymatic assay
TSH	0.340-6.050 μIU/L	Electrochemiluminescence immunoassay method
FT3	2.39-4.26 pg/ml	Electrochemiluminescence immunoassay method
FT4	0.58-1.64 ng/dl	Electrochemiluminescence immunoassay method
TT3	0.75-1.58 ng/ml	Electrochemiluminescence immunoassay method
TT4	6.09-12.23μg/dl	Electrochemiluminescence immunoassay method
Hemoglobin	115-150 g/L	Sodium dodecyl sulfate method
Folate	7.0-45.1 nmol/L	Chemiluminescent method
Vitamin B12	133-675 pmol/L	Chemiluminescent method
Ferritin	11.0-306.8 ng/ml	Chemiluminescent method
Estradiol	early follicular phase: 55.64-469.06 pmol/L;<br>mid follicular phase: 72.88-543.64 pmol/L;<br>peak ovulation phase: 107.97-1624.42 pmol/L;<br>mid luteal phase: 111.35-1006.46 pmol/L;<br>menopause: ≤92.12 pmol/L	Chemiluminescent method
Testosterone	0.35-2.60 nmol/L	Chemiluminescent method
FSH,	follicular phase: 3.30-8.60 mIU/ml;<br>ovulatory period: 3.30-22.20 mIU/ml;<br>luteal phase: 0.70-5.00 mIU/ml;<br>menopause: 21.00-104.00 mIU/ml	Chemiluminescent method
LH	follicular phase: 1.50-12.00 mIU/ml;<br>ovulatory period: 23.00-109.00 mIU/ml;<br>luteal phase: 1.00-5.00 mIU/ml;<br>menopause:10.90-58.60 mIU/ml	Chemiluminescent method
Prolactin	premenopause: 70.808-566.464 mIU/L;<br>menopause: 58.088-416.368 mIU/L	Chemiluminescent method

Abbreviations: ALT, alanine transpeptidase; AST, aspartate transaminase; C-P, fasting C-peptide; FPG, fasting plasma glucose; FSH, follicle stimulating hormone; FT3, free triiodothyronine; FT4, free thyroxine; GGT, gamma glutamyl transferase; HbA1c, glycosylated hemoglobin; HDL-C, high-density lipoprotein cholesterol; Ins, fasting insulin; LDL-C, low-density lipoprotein cholesterol; LH, luteinizing hormone; LP(a), lipoprotein (a); TC, total cholesterol; TSH, thyroid-stimulating hormone; TT3, total triiodothyronine; TT4, total thyroxine; UA, uric acid.

Table S7. Proportions of missing data and comparisons between pre- and post-imputation in the derivation cohort and external validation cohort

Characteristics	Derivation cohort (n=1094)				External validation cohort (n=218)			
	Percentage of missing data, n (%)	Original data	Multiple imputation	P-value	Percentage of missing data, n (%)	Original data	Multiple imputation	P-value
Waist circumference, cm	96 (8.8)	116.0 [107.5, 129.0]	116.0 [107.0, 128.0]	0.609	24 (11.0)	114.0 [105.0, 128.0]	114.0 [105.0, 128.0]	0.979
Hip circumference, cm	147 (13.4)	121.0 [114.0, 132.0]	121.0 [114.0, 131.0]	0.807	24 (11.0)	119.0 [110.0, 132.0]	120.0 [110.0, 132.0]	0.960
Smoking history, n (%)	NA	NA	NA	NA	1 (0.5)	28 (12.9)	28 (12.8)	1.000
Alcohol consumption, n (%)	1 (0.1)	116 (10.6)	116 (10.6)	1.000	1 (0.5)	17 (7.8)	17 (7.8)	1.000
Hypertension, n (%)	7 (0.6)	434 (39.9)	437 (39.9)	1.000	NA	NA	NA	NA
T2DM, n (%)	1 (0.1)	532 (48.7)	533 (48.7)	1.000	NA	NA	NA	NA
FPG, mmol/L	2 (0.2)	5.8 [5.07, 7.20]	5.8 [5.1, 7.2]	0.996	23 (10.6)	5.4 [4.9, 6.8]	5.4 [4.9, 6.7]	0.725
Ins, µIU/ml	40 (3.7)	27.3 ± 19.6	27.2 ± 19.4	0.914	18 (8.3)	29.9 ± 18.3	30.0 ± 19.1	0.945
C-P, ng/ml	41 (3.7)	4.4 ± 3.0	4.4 ± 3.0	0.869	17 (7.8)	4.3 ± 1.8	4.3 ± 1.8	0.900
HbA1c, %	5 (0.5)	6.0 [5.5, 7.1]	6.0 [5.5, 7.1]	0.941	23 (10.6)	5.9 [5.5, 6.6]	5.9 [5.5, 6.6]	0.901
ALT, IU/L	4 (0.4)	38.0 [25.0, 66.0]	38.0 [25.0, 66.0]	0.958	1 (0.5)	33.0 [19.4, 66.5]	32.5 [19.3, 66.4]	0.951
AST, IU/L	3 (0.3)	24.0 [17.0, 36.0]	24.0 [17.0, 36.0]	0.963	NA	NA	NA	NA
GGT, IU/L	10 (0.9)	43.7 ± 38.8	43.7 ± 38.7	0.970	NA	NA	NA	NA
UA, µmol/L	NA	NA	NA	NA	12 (5.5)	405.7 ± 97.3	401.9 ± 96.7	0.689
TC, mmol/L	1 (0.1)	4.9 ± 1.0	4.9 ± 1.0	0.970	14 (6.4)	5.0 ± 1.0	5.0 ± 1.0	0.715
Triglyceride, mmol/L	1 (0.1)	2.2 ± 2.1	2.2 ± 2.1	0.991	2 (0.9)	2.2 ± 2.3	2.2 ± 2.3	0.997
HDL-C, mmol/L	1 (0.1)	1.0 ± 0.2	1.0 ± 0.2	0.989	2 (0.9)	1.1 ± 0.3	1.1 ± 0.3	0.988
LDL-C, mmol/L	1 (0.1)	3.0 ± 0.7	3.0 ± 0.7	0.995	2 (0.9)	2.9 ± 0.8	2.9 ± 0.8	0.951
LP(a), mg/L	78 (7.1)	66.4 [35.2, 142.6]	66.2 [35.2, 141.7]	0.984	NA	NA	NA	NA
Calcium, mmol/L	7 (0.6)	2.3 ± 0.1	2.3 ± 0.1	0.889	21 (9.6)	2.3 ± 0.2	2.3 ± 0.2	0.861
Creatinine, µmol/L	NA	NA	NA	NA	1 (0.5)	60.1 ± 15.4	60.0 ± 15.3	0.980
TSH, uIU/L	12 (1.1)	2.8 ± 2.6	2.8 ± 2.6	0.978	15 (6.9)	2.4 ± 1.4	2.4 ± 1.4	0.995
FT3, pg/ml	8 (0.7)	3.3 ± 0.5	3.3 ± 0.5	0.980	16 (7.3)	3.8 ± 1.1	3.8 ± 1.0	0.906
FT4, ng/dl	9 (0.8)	1.1 ± 0.3	1.1 ± 0.3	0.971	16 (7.3)	1.1 ± 0.4	1.1 ± 0.4	0.983
TT3, ng/ml	11 (1.0)	1.2 ± 0.2	1.2 ± 0.2	0.908	NA	NA	NA	NA
TT4, ug/dl	10 (0.9)	8.3 ± 1.8	8.4 ± 1.8	0.940	NA	NA	NA	NA
Hemoglobin, g/L	4 (0.4)	136.7 ± 17.5	136.7 ± 17.5	0.998	1 (0.5)	141.5 ± 16.4	141.4 ± 16.4	0.957
Folate, nmol/L	9 (0.8)	19.0 [13.2, 29.3]	19.0 [13.2, 29.3]	0.961	NA	NA	NA	NA

Vitamin B12, pmol/L	7 (0.6)	222.0 [167.9, 293.1]	222.1 [168.0, 293.0]	0.977	NA	NA	NA	NA
Ferritin, ng/ml	8 (0.7)	80.8 [39.8, 162.4]	80.9 [39.6, 163.4]	0.975	NA	NA	NA	NA
Estradiol, pmol/L	15 (1.4)	219.0 [133.0, 525.5]	218.4 [132.2, 525.5]	0.984	17 (7.8)	137.6 [99.6, 237.2]	134.7 [101.9, 236.0]	0.941
Testosterone, nmol/L	15 (1.4)	3.9 [1.8, 8.5]	3.9 [1.8, 8.5]	0.958	16 (7.3)	2.2 [1.5, 5.8]	2.2 [1.5, 5.9]	0.786
FSH, mIU/ml	15 (1.4)	5.0 [3.5, 6.9]	5.1 [3.5, 6.9]	0.960	NA	NA	NA	NA
LH, mIU/ml	15 (1.4)	5.1 [3.2, 8.0]	5.1 [3.2, 8.0]	0.955	NA	NA	NA	NA
Prolactin, mIU/L	16 (1.5)	426.4 [259.6, 4257.7]	426.7 [259.1, 4272.8]	0.969	NA	NA	NA	NA

The categorical variables of the cohort data were expressed as n (%), and continuous variables with normal distribution were expressed as mean ± standard deviation, while medians [quartiles] were used for variables that did not satisfy normal distribution.

The p-values refer to the comparison between pre- and post-imputation data with the use of Student t test or the Mann–Whitney U test for continuous variables and Fisher exact test or Pearson x^2 test.

Abbreviations: ALT, alanine transpeptidase; AST, aspartate transaminase; C-P, fasting C-peptide; FPG, fasting plasma glucose; FSH, Follicle stimulating hormone; FT3, free triiodothyronine; FT4, free thyroxine; GGT, gamma glutamyl transferase; HbA1c, glycosylated hemoglobin; HDL-C, high-density lipoprotein cholesterol; Ins, fasting insulin; LDL-C, low-density lipoprotein cholesterol; LH, luteinizing hormone; LP(a), lipoprotein (a); NA, not applicable; TC, total cholesterol; TSH, thyroid-stimulating hormone; TT3, total triiodothyronine; TT4, total thyroxine; T2DM, type 2 diabetes mellitus; UA, uric acid.

Table S8. The hyperparameters for six models

Models	Code and hyperparameters
Logistic Regression	model = LogisticRegression (penalty='l1', C=0.3, solver='liblinear', random_state=1386)
Naive Bayes	model = GaussianNB ( )
Neural net	model = MLPClassifier (activation='tanh', alpha=0.0001, hidden_layer_sizes=(50, 50), learning_rate='constant', solver='sgd', random_state=1386)
Quadratic Discriminant Analysis	model = QuadraticDiscriminantAnalysis (priors=None, reg_param=0.5, tol=0.001)
Support Vector Machine	model = SVC (C=1, gamma=0.01, kernel='linear', probability=True, random_state=1386)
Extreme Gradient Boosting	model = XGBClassifier (n_estimators=300, max_depth=7, learning_rate=0.05, subsample=0.6, gamma=13, reg_alpha=0.05, reg_lambda=0.7, colsample_bytree=1, min_child_weight=3, random_state=1386)

Table S9. The information of expert board

Human experts	Years of experience	Centers
Junior expert 1	2	Department of General Surgery & Obesity and Metabolic Disease Center, China-Japan Friendship Hospital
Junior expert 2	2	Department of General Surgery & Obesity and Metabolic Disease Center, China-Japan Friendship Hospital
Junior expert 3	4	Department of General Surgery & Obesity and Metabolic Disease Center, China-Japan Friendship Hospital
Middle-level expert 1	7	Department of General Surgery & Obesity and Metabolic Disease Center, China-Japan Friendship Hospital
Middle-level expert 2	7	Department of General Surgery & Obesity and Metabolic Disease Center, China-Japan Friendship Hospital
Middle-level expert 3	7	Department of General Surgery & Obesity and Metabolic Disease Center, China-Japan Friendship Hospital
Senior expert 1	12	Department of Gastrointestinal Metabolic Surgery, Zhengzhou Central Hospital
Senior expert 2	13	Department of Endocrinology, The Second Hospital of Hebei Medical University
Senior expert 3	15	Department of General Surgery & Obesity and Metabolic Disease Center, China-Japan Friendship Hospital
Senior expert 4	19	Department of Gastrointestinal Surgery, The Second Affiliated Hospital of Wenzhou Medical University

Figure S1. Internal validation of XGB model

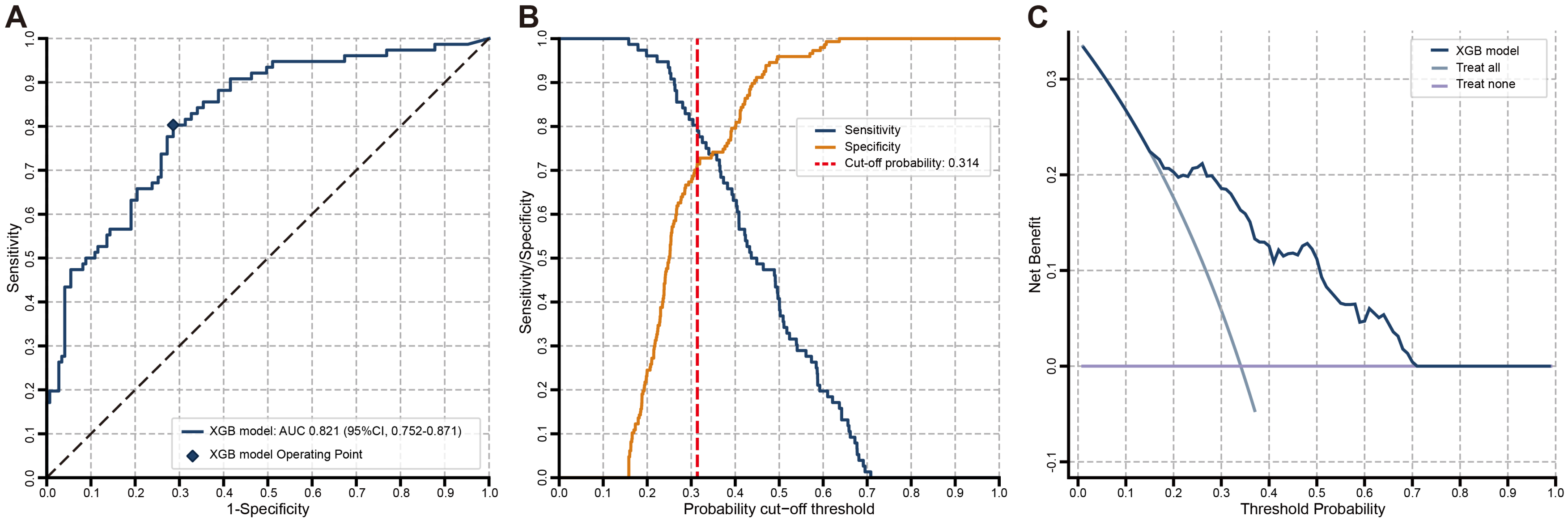

(A) Discrimination of the XGB model by ROC curve analysis. (B) Sensitivity and specificity versus cut-off probability plot of the XGB model. Decreasing sensitivity and increasing specificity are shown for increasing probability thresholds for SCR. (C) Decision curve analysis of the XGB model for clinical utility. Abbreviation: AUC, area under the receiver operating characteristic curve; SCR, suboptimal clinical response.

Figure S2. Visualization of the prediction by 10 human experts

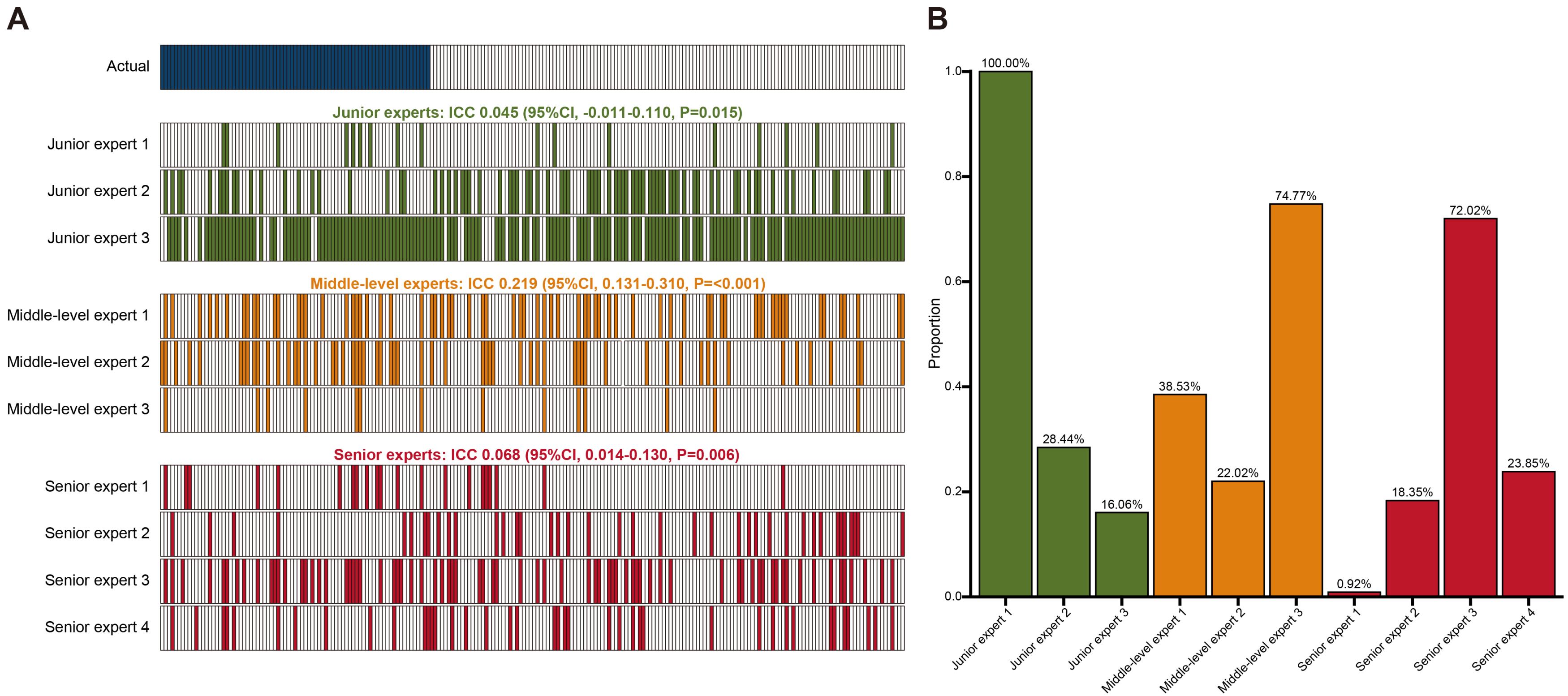

(A) Each row represents predictions made by a human expert and each small box represents a patient, with the filled color indicating the occurrence (predicted as) SCR, and the blank representing OCR. The ICC was used to evaluate the reliability of estimates among the experts. (B) The proportion of patients deemed to have uncertain predictions by ten human experts.

Abbreviation: ICC, Interclass Correlation Coefficient; OCR, optimal clinical response; SCR, suboptimal clinical response.

Figure S3. Performance of the XGB model and its comparison with human experts in the external validation cohort 1

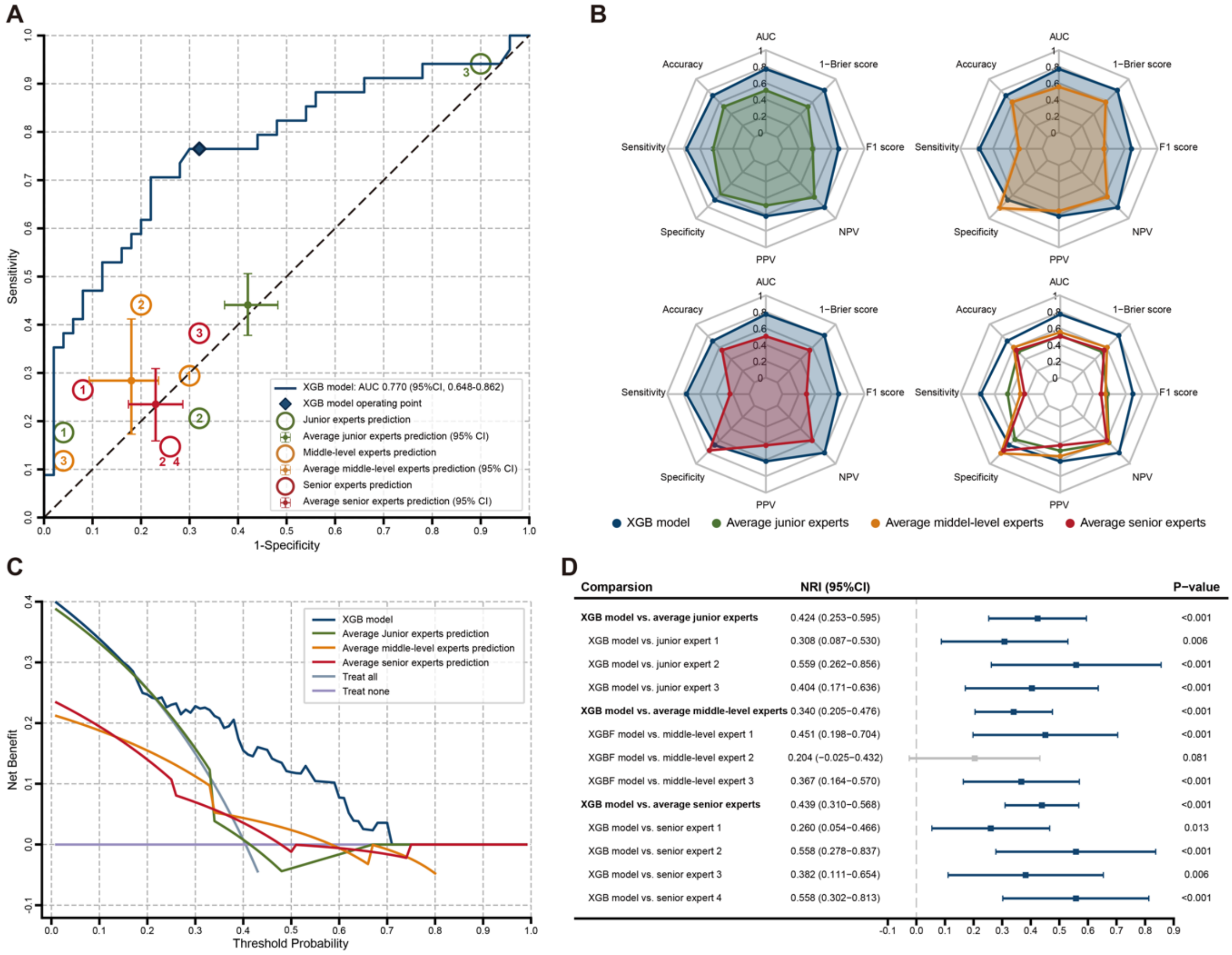

(A) Discrimination of the XGB model by ROC curve analysis. The operating point was determined by the Youden index. Each small colored circle represents the sensitivity and specificity of an individual expert, with error bars illustrating the 95% CI for the average sensitivity and specificity of three groups of experts. (B) The performance of the XGB model was compared with that of three groups of experts across multiple metrics. (C) Decision curve analysis comparing the net benefit of the XGB model with that of three groups of experts across a range of threshold probabilities. (D) The categorical NRI was calculated to assess the improvement in predictive accuracy of the model compared with expert judgment.

Abbreviation: AUC, area under the receiver operating characteristic curve; CI, Confidence interval; NPV, negative predictive value; NRI, net reclassification index; PPV, positive predictive value.

Figure S4. Performance of the XGB model and its comparison with human experts in the external validation cohort 2

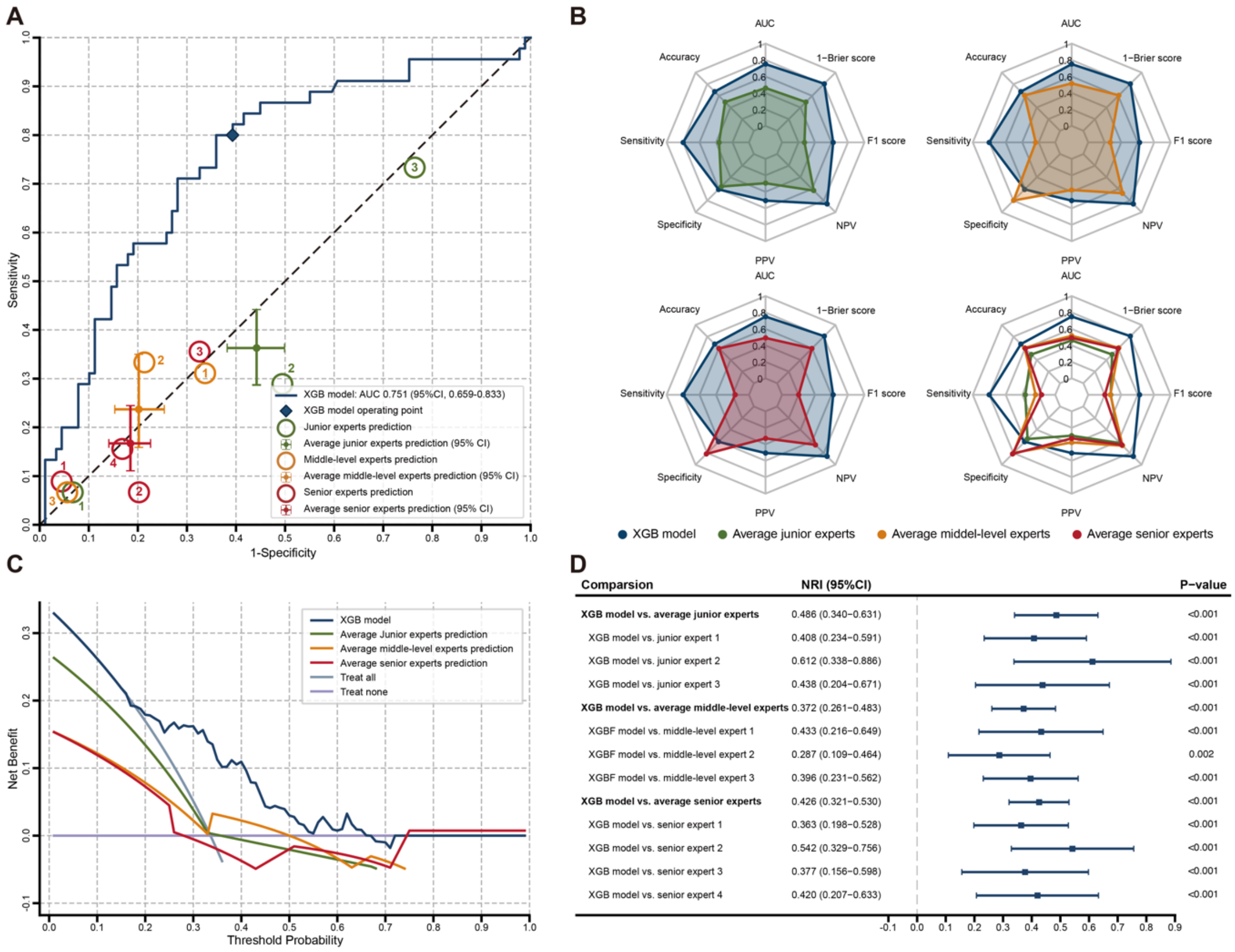

(A) Discrimination of the XGB model by ROC curve analysis. The operating point was determined by the Youden index. Each small colored circle represents the sensitivity and specificity of an individual expert, with error bars illustrating the 95% CI for the average sensitivity and specificity of three groups of experts. (B) The performance of the XGB model was compared with that of three groups of experts across multiple metrics. (C) Decision curve analysis comparing the net benefit of the XGB model with that of three groups of experts across a range of threshold probabilities. (D) The categorical NRI was calculated to assess the improvement in predictive accuracy of the model compared with expert judgment.

Abbreviation: AUC, area under the receiver operating characteristic curve; CI, Confidence interval; NPV, negative predictive value; NRI, net reclassification index; PPV, positive predictive value.

Figure S5. Performance of the XGB model and its comparison with human experts in patients considered challenging by the experts

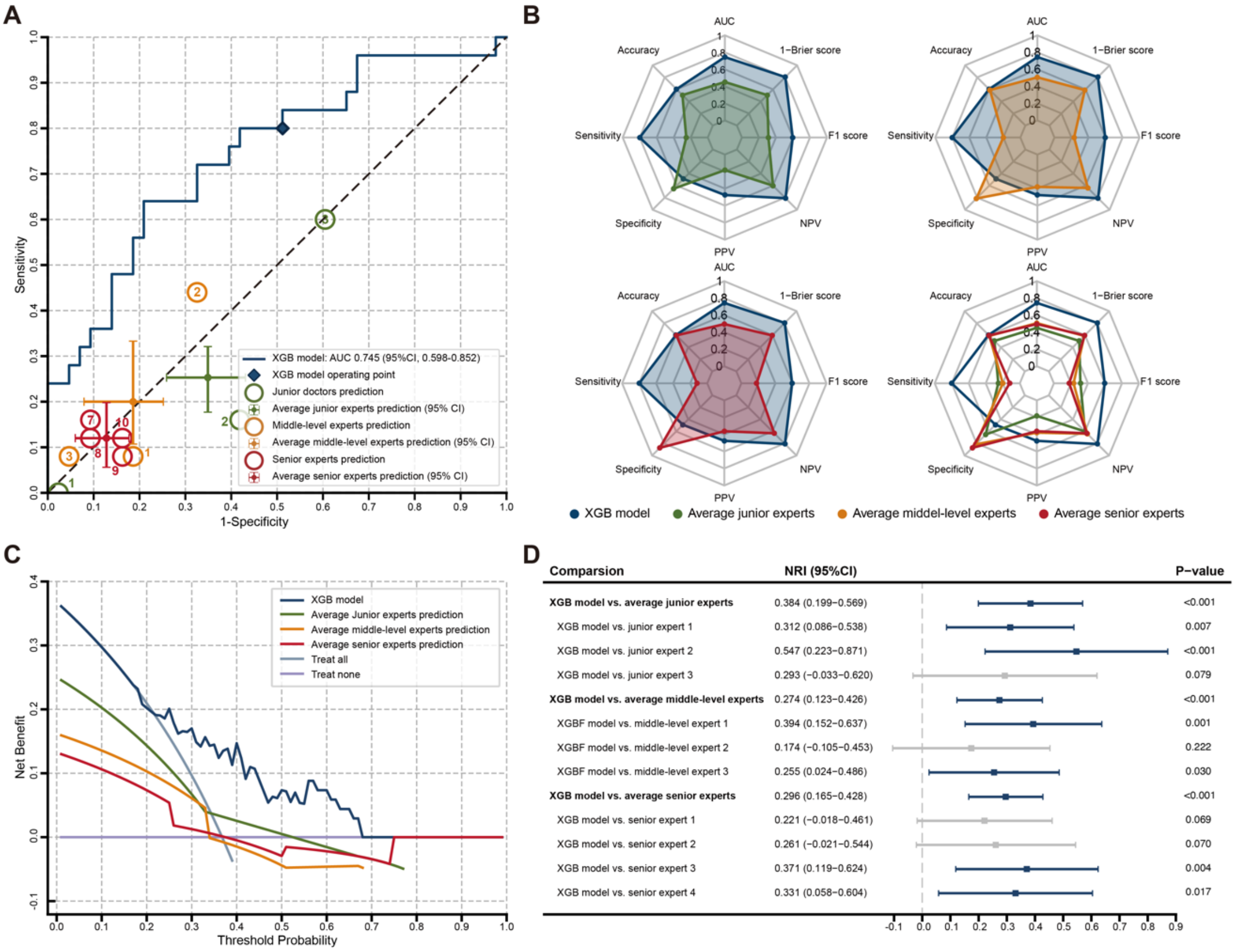

(A) Discrimination of the XGB model by ROC curve analysis. The operating point was determined by the Youden index. Each small colored circle represents the sensitivity and specificity of an individual expert, with error bars illustrating the 95% CI for the average sensitivity and specificity of three groups of experts. (B) The performance of the XGB model was compared with that of three groups of experts across multiple metrics. (C) Decision curve analysis comparing the net benefit of the XGB model with that of three groups of experts across a range of threshold probabilities. (D) The categorical NRI was calculated to assess the improvement in predictive accuracy of the model compared with expert judgment.

Abbreviation: AUC, area under the receiver operating characteristic curve; CI, Confidence interval; NPV, negative predictive value; NRI, net reclassification index; PPV, positive predictive value.

Figure S6. SHAP dependence plot for each feature in the XGB model

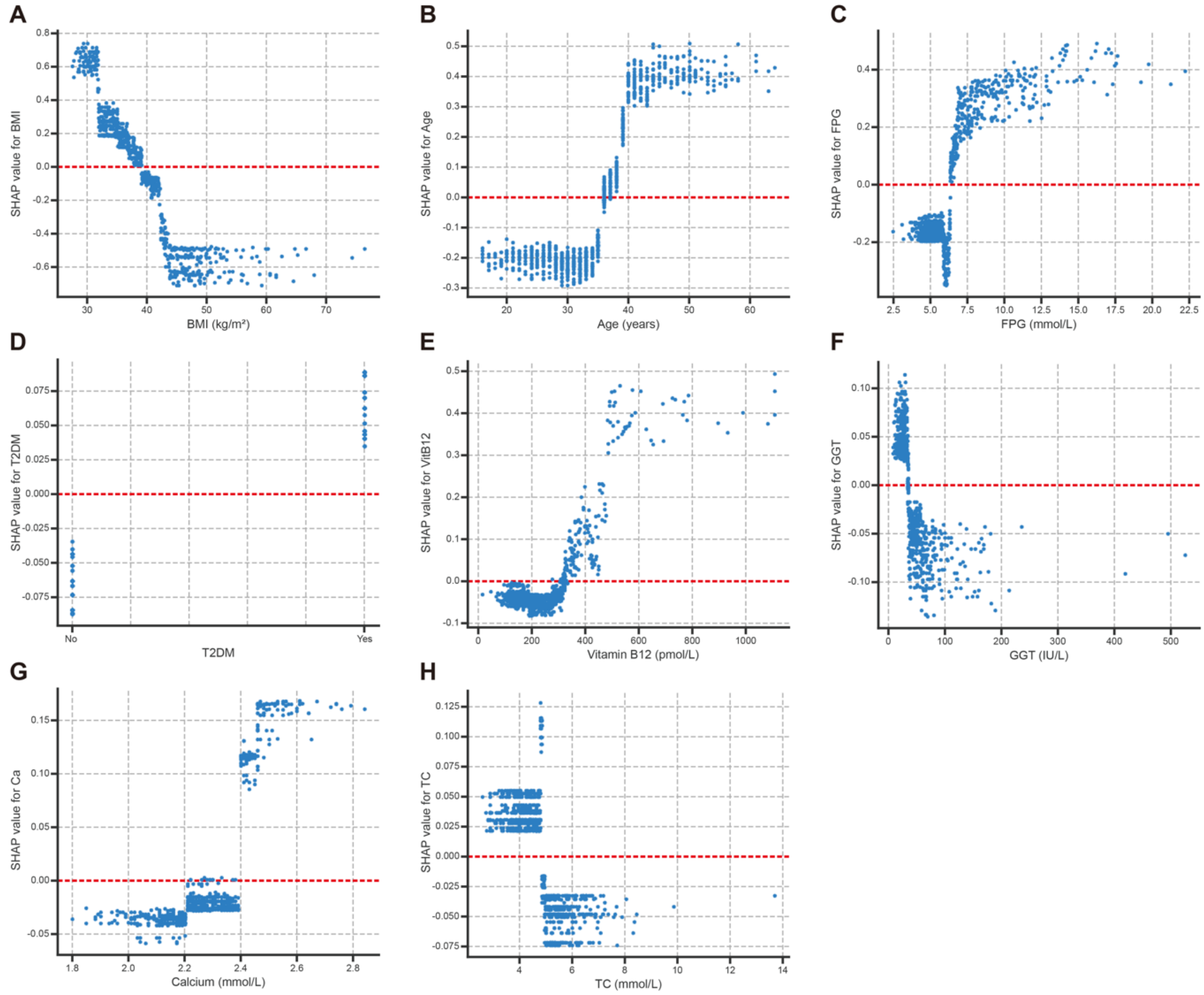

Each dependence plot demonstrates how a single feature influences the prediction output of XGB model, with each dot representing an individual patient. The SHAP values are plotted on the y-axis, while the actual feature values are shown on the x-axis. SHAP values exceeding zero for specific features drive the output of XGB model towards the 'SCR' classification.

Abbreviation: BMI,4; FPG, fasting plasma glucose; GGT, gamma glutamyl transferase; SCR, suboptimal clinical response; SHAP, SHapley Additive exPlanation; TC, total cholesterol; T2DM, type 2 diabetes mellitus.

Figure S7. Comparison of the performance of six candidate models

A

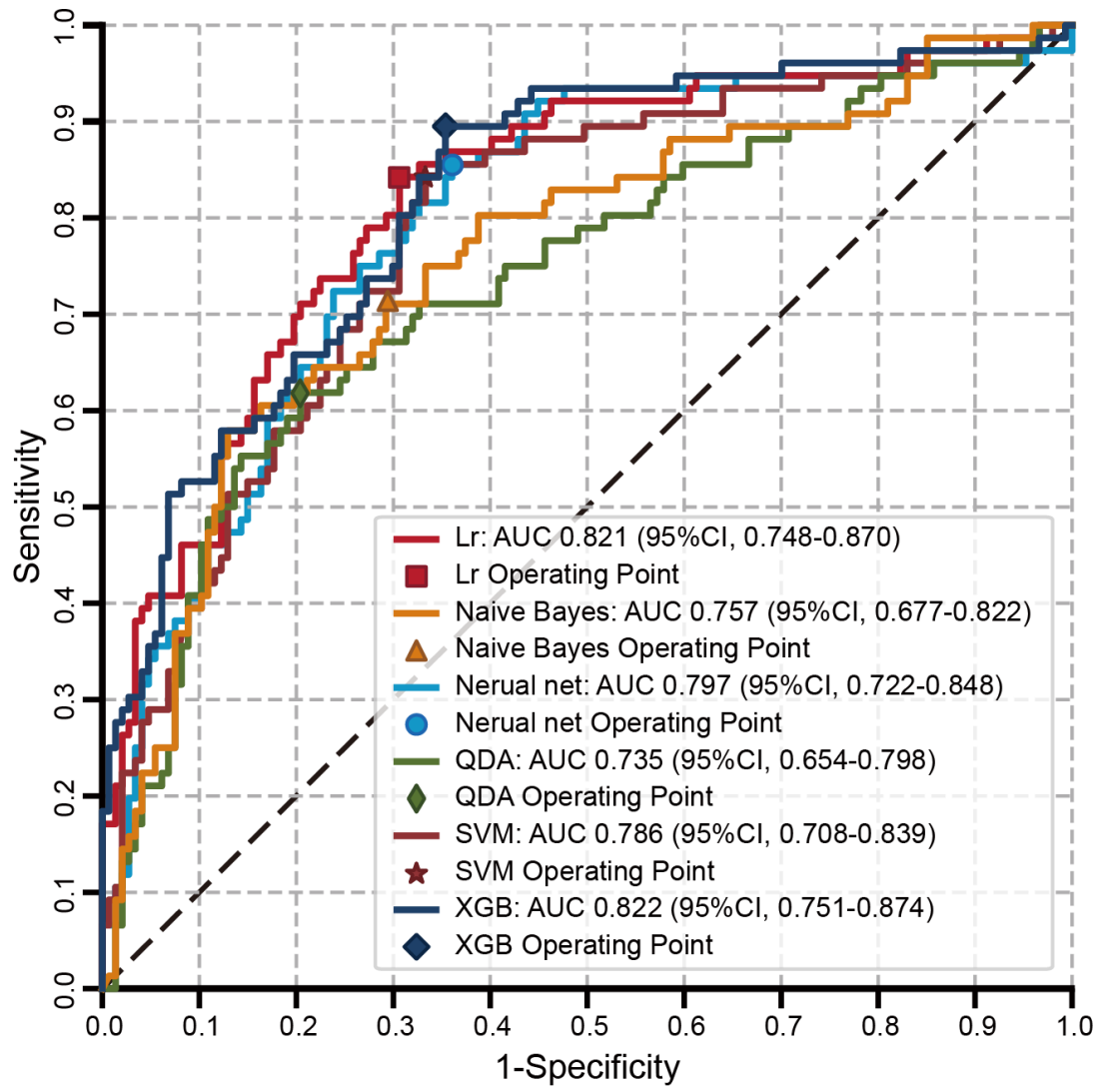

B

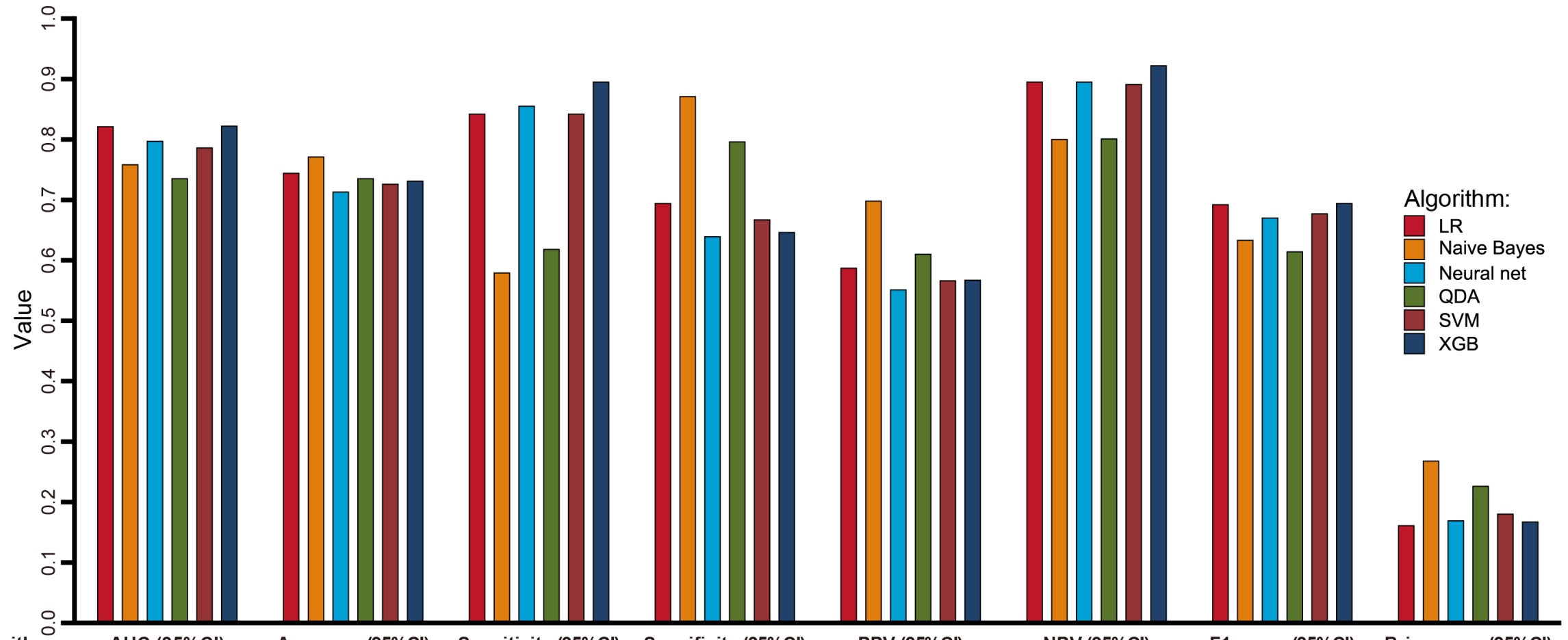

Algorithms	AUC (95%CI)	Accuracy (95%CI)	Sensitivity (95%CI)	Specificity (95%CI)	PPV (95%CI)	NPV (95%CI)	F1 score (95%CI)	Brier score (95%CI)
LR	0.821 (0.748-0.870)	0.744 (0.682-0.794)	0.842 (0.743-0.914)	0.694 (0.616-0.766)	0.587 (0.507-0.683)	0.895 (0.830-0.941)	0.692 (0.610-0.762)	0.161 (0.143-0.190)
Naive Bayes	0.757 (0.677-0.822)	0.771 (0.709-0.821)	0.579 (0.468-0.685)	0.871 (0.813-0.917)	0.698 (0.578-0.803)	0.800 (0.731-0.853)	0.633 (0.531-0.715)	0.268 (0.222-0.316)
Neural net	0.797 (0.722-0.848)	0.713 (0.646-0.767)	0.855 (0.760-0.923)	0.639 (0.556-0.713)	0.551 (0.463-0.644)	0.895 (0.821-0.944)	0.670 (0.586-0.742)	0.169 (0.148-0.199)
QDA	0.735 (0.654-0.798)	0.735 (0.664-0.785)	0.618 (0.500-0.720)	0.796 (0.727-0.857)	0.610 (0.488-0.716)	0.801 (0.731-0.859)	0.614 (0.519-0.700)	0.226 (0.189-0.270)
SVM	0.786 (0.708-0.839)	0.726 (0.664-0.780)	0.842 (0.736-0.906)	0.667 (0.578-0.738)	0.566 (0.476-0.657)	0.891 (0.817-0.940)	0.677 (0.595-0.752)	0.180 (0.162-0.207)
XGB	0.822 (0.751-0.874)	0.731 (0.668-0.785)	0.895 (0.819-0.950)	0.646 (0.571-0.724)	0.567 (0.478-0.655)	0.922 (0.857-0.965)	0.694 (0.615-0.758)	0.167 (0.148-0.191)

(A) Six candidate models were evaluated in the testing set, and the ROC curves were plotted. The operating point was determined by the Youden index, representing the maximum sum of Sensitivity and Specificity. (B) The performance of six candidate models was evaluated across complementary evaluation metrics and summarized in the bar chart.

Abbreviations: LR, Logistic Regression; NPV, negative predictive value; PPV, positive predictive value; QDA, Quadratic Discriminant Analysis; SVM, Support Vector Machine; XGB, Extreme Gradient Boosting.

Figure S8. The feature importance rankings across the six models

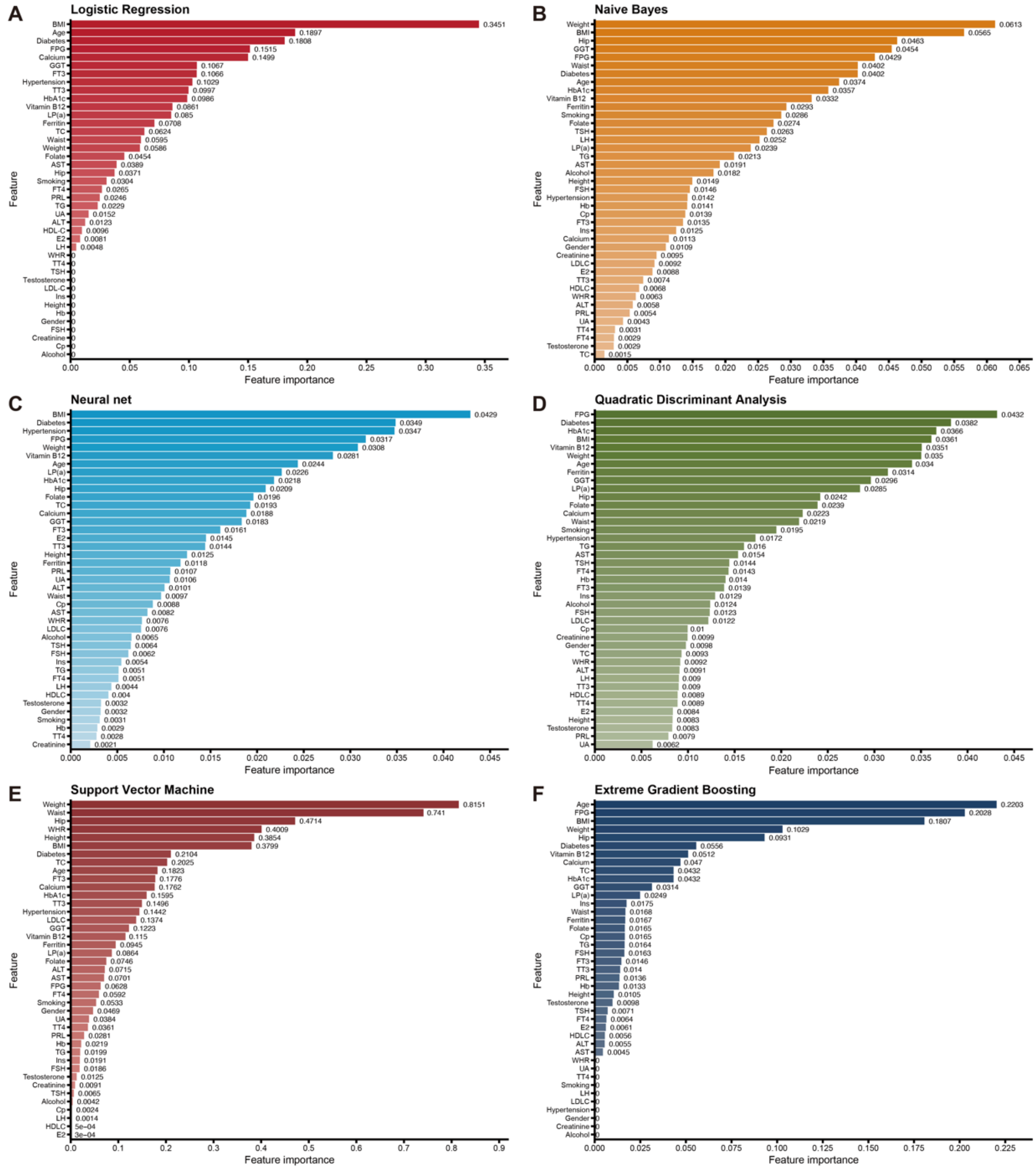

SHAP was used to compute feature importance for each model, with features ranked in descending order of importance within each model. Figures (A-F) represent Logistic Regression, Naive Bayes, Neural net, Quadratic Discriminant Analysis, Support Vector Machine, and Extreme Gradient Boosting, respectively. Abbreviations: ALT, alanine transpeptidase; AST, aspartate transaminase; BMI, body mass index; Ins, fasting insulin; Ca, calcium; C-P, fasting C-peptide; FPG, fasting plasma glucose; FSH, follicle stimulating hormone; FT3, free triiodothyronine; FT4, free thyroxine; GGT, gamma glutamyl transferase; HbA1c, glycated hemoglobin; HDL-C, high-density lipoprotein cholesterol; Ins, fasting insulin; LDL-C, low-density lipoprotein cholesterol; LH, luteinizing hormone; LP(a), lipoprotein (a); OCR, optimal clinical response; SCR, suboptimal clinical response; TC, total cholesterol; TSH, thyroid-stimulating hormone; TT3, total triiodothyronine; TT4, total thyroxine; T2DM, type 2 diabetes mellitus; UA, uric acid; VitB12, vitamin B12.

Figure S9. The process of features selection

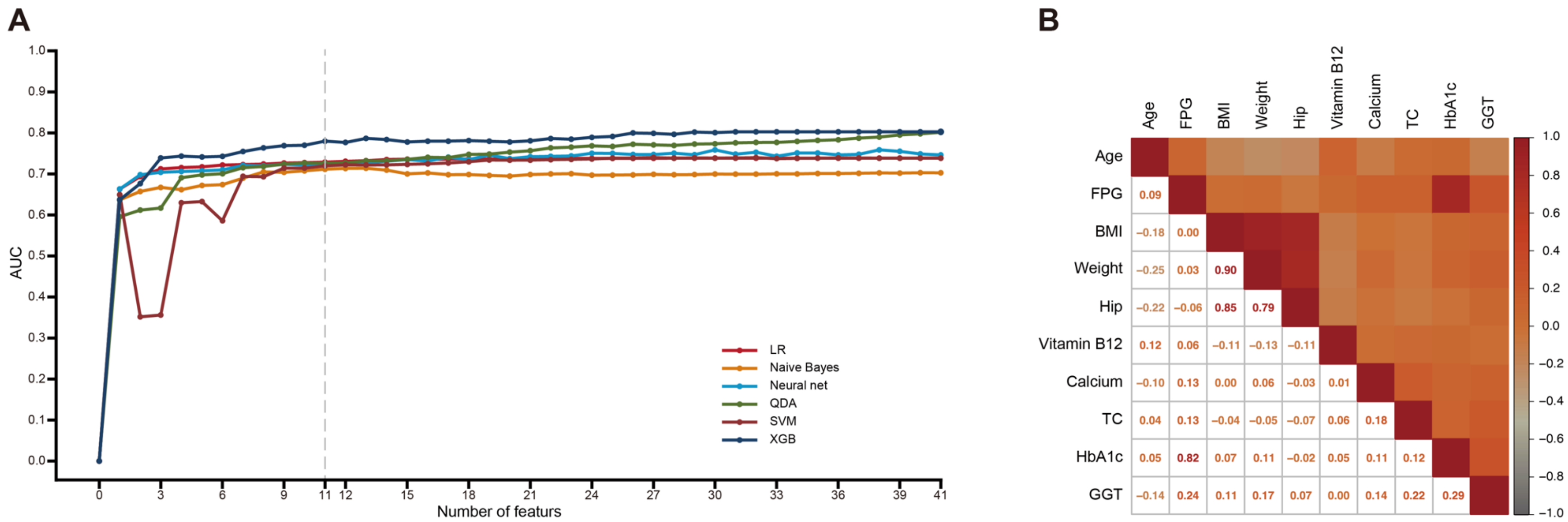

(A) The figure illustrates the relationship between AUC variation and the number of features incorporated in the models across six models. (B) The heatmap illustrates the relationships among the retained continuous features, with correlations measured using the Pearson correlation coefficient.

Abbreviation: AUC, area under the receiver operating characteristic curve; BMI, body mass index; FPG, fasting plasma glucose; GGT, gamma glutamyl transferase; HbA1c, glycated hemoglobin; LR, Logistic Regression; QDA, Quadratic Discriminant Analysis, SVM, Support Vector Machine; TC, total cholesterol; XGB, Extreme Gradient Boosting.
